# Genome-wide characterization and expression analysis of citrus NUCLEAR FACTOR-Y (NF-Y) transcription factors identified a novel *NF-YA* gene involved in drought-stress response and tolerance

**DOI:** 10.1371/journal.pone.0199187

**Published:** 2018-06-15

**Authors:** Suzam L. S. Pereira, Cristina P. S. Martins, Aurizangela O. Sousa, Luciana R. Camillo, Caroline P. Araújo, Grazielle M. Alcantara, Danielle S. Camargo, Luciana C. Cidade, Alex-Alan F. de Almeida, Marcio G. C. Costa

**Affiliations:** Centro de Biotecnologia e Genética, Departamento de Ciências Biológicas, Universidade Estadual de Santa Cruz, Ilhéus, Bahia, Brazil; Institute of Genetics and Developmental Biology Chinese Academy of Sciences, CHINA

## Abstract

Nuclear factor Y (NF-Y) is a ubiquitous transcription factor found in eukaryotes. It is composed of three distinct subunits called NF-YA, NF-YB and NF-YC. NF-Ys have been identified as key regulators of multiple pathways in the control of development and tolerance to biotic and abiotic factors. The present study aimed to identify and characterize the complete repertoire of genes coding for NF-Y in citrus, as well as to perform the functional characterization of one of its members, namely *CsNFYA5*, in transgenic tobacco plants. A total of 22 genes coding for NF-Y were identified in the genomes of sweet orange (*Citrus sinensis*) and Clementine mandarin (*C*. *clementina*), including six CsNF-YAs, 11 CsNF-YBs and five CsNF-YCs. Phylogenetic analyses showed that there is a NF-Y orthologous in the Clementine genome for each sweet orange NF-Y gene; this was not observed when compared to *Arabidopsis thaliana*. CsNF-Y proteins shared the same conserved domains with their orthologous proteins in other organisms, including mouse. Analysis of gene expression by RNA-seq and EST data demonstrated that *CsNF-Y*s have a tissue-specific and stress inducible expression profile. qRT-PCR analysis revealed that *CsNF-YA5* exhibits differential expression in response to water deficit in leaves and roots of citrus plants. Overexpression of *CsNF-YA5* in transgenic tobacco plants contributed to the reduction of H_2_O_2_ production under dehydration conditions and increased plant growth and photosynthetic rate under normal conditions and drought stress. These biochemical and physiological responses to drought stress promoted by *CsNF-YA5* may confer a productivity advantage in environments with frequent short-term soil water deficit.

## Introduction

Nuclear Factor Y (NF-Y), also known as heme activator protein (HAP) or CCAAT-binding factor (CBF), is a heterotrimeric complex transcription factor composed of three distinct subunits called NF-YA (HAP2), NF-YB (HAP3/CBF-A) and NF-YC (HAP5/CBF-C). NF-Ys are evolutionarily conserved in eukaryotes, with each subunit encoded by a single gene in yeasts and animals, and by gene families comprising between eight and 39 members in plants [1]. The current complex assembly model for NF-Y suggests that NF-YB and C initially form a dimer in the cytoplasm, and subsequently translocated to the cell nucleus where they interact with NF-YA to form a heterotrimer complex [2,3]. NF-YA has a characteristically high affinity to and specificity for the CCAAT box, a *cis*-acting element present in approximately 25% of eukaryotic gene promoters [4].

NF-Ys have emerged as important regulators of various developmental processes and stress tolerance in plants. *Arabidopsis* NF-YA genes have been shown to regulate gametogenesis, embryogenesis, seed development and flowering [5–7], while in leguminous plants they have been reported as key regulators of organogenesis and development of symbiotic root nodules [8–11]. NF-YB has been implicated in the regulation of embryogenesis, seed and nodule development [9,12–14], flowering time [15–18], chloroplast biogenesis [19], cell proliferation and endosperm development [20], root elongation [21], photosynthesis [22] and photomorphogenesis [23]. NF-YCs have been likewise found to be involved in the control of flowering time [15,17,18,24], symbiotic nodule development [8,25], root growth [25], photosynthesis [26] and photomorphogenesis [27,28].

The overexpression of the drought-induced and guard cell highly expressed *Arabidopsis NF-YA5* (*AtNF-YA5*) gene improved drought tolerance and reduced leaf water loss, while *nfya5* knockout mutants and plants overexpressing the microRNA miR169a showed enhanced leaf water loss and increased sensitive to drought stress in comparison with wild-type (WT) plants [4]. Microarray analysis indicated that *AtNF-YA5* plays a role in the induction of expression for a number of stress-responsive genes, such as those involved in oxidative stress responses [4]. *Arabidopsis* transgenic plants overexpressing the soybean *NF-YA3* gene have also shown reduced water loss in leaves and enhanced drought tolerance, with an increased expression of ABA-biosynthesis (*ABA1* and *ABA2*), ABA signaling (*ABI1* and *ABI2*) and stress-responsive (*RD29A* and *CBF3*) genes [29]. Transgenic rice plants overexpressing the *OsHAP2E* gene showed tolerance to drought and salt stresses and resistance to *Magnaporthe oryzae* and *Xanthomonas oryzae* infections, as well as higher chlorophyll contents, photosynthetic rates and number of tillers than WT plants [30]. Microarray analysis showed that *OsHAP2E* up-regulated a number of defense (e.g. chitinase, PBZ1, beta-1,3-glucanase and thaumatin-like protein) and oxidative stress-related (glutathione S-transferase) genes [30]. Overexpression of drought-inducible *NF-YB* and *NF-YC* genes also has been shown to improve the drought stress tolerance in *Arabidopsis*, maize, poplar and rice [31–34].

*Citrus* is an important genus that includes several cultivated species, such as *C*. *sinensis* (sweet oranges), *C*. *reticulata* (mandarins and tangerines), *C*. *limon* (lemons/limes), *C*. *grandis* (pummelos) and *C*. *paradisi* (grapefruits). *Citrus* cultivation is mainly located in semiarid regions of the world, where the trees constantly face problems related to water scarcity and drought stress, which are even more pronounced by the use of saline waters for irrigation. In light of this, efforts have been made to identify drought-responsive genes in citrus for use in rootstock breeding programs aiming to obtain drought-tolerant plants. The availability of the reference genome sequences for *C*. *sinensis* and *C*. *clementina* [35,36], as well as a rich database (HarvEST: Citrus) of expressed sequence tags (ESTs) for different tissues of several *Citrus* species and the related genus *Poncirus* under distinct treatments, now provides the possibility to identify and characterize potential candidate genes for drought response and tolerance in citrus and to accelerate the generation of drought-tolerant citrus rootstocks.

Considering the important role of NF-Ys in mediating drought tolerance in plants, in the present study we have characterized the complete set of citrus *NF-Y* genes. We further characterized functionally a citrus drought-responsive *NF-YA* family member, with no close homologs in *Arabidopsis*, through its overexpression in transgenic tobacco plants.

## Materials and methods

### Plant material and stress treatments

Two-year-old sweet orange (*Citrus sinensis* L. Osb. var. ‘Westin’) plants grafted on Rangpur lime (*C*. *limonia* Osbeck) were used in the drought stress experiment. The experiment was carried out under greenhouse conditions, with the plants grown in plastic pots of 45L containing a mixture of Oxisol and washed sand (3:1 ratio) and subjected to control (leaf predawn water potential between -0.2 to -0.4 MPa) or drought stress (leaf predawn water potential of -1.5 MPa) treatments, as previously described [37].

The plants of *Nicotiana tabacum* cv. Havana used in *Agrobacterium*-mediated genetic transformation experiments, were derived from pre-established cultures kept in the Tissue Culture Laboratory of the Center for Biotechnology and Genetics (CBG) of the State University of Santa Cruz (UESC). The stress tolerance of wild-type (WT) and transgenic tobacco plants was examined by the leaf-disc dehydration tolerance assay, *in vitro* drought stress tolerance assay and soil dry-down experiment under greenhouse conditions, as previously described [38]. In brief, uniform samples of leaf discs were extracted from mature, fully expanded leaves of adult plants of WT and transgenic (T_0_ generation) tobacco kept in a greenhouse; this was done with the aid of a 1-cm diameter punch, avoiding the region of the central vein. Leaf discs were then subjected to dehydration at room temperature for 210 min, and fresh weight measurements were performed every 30 min using an analytical scale. The rate of dehydration was determined as the percentage of FW loss versus the initial fresh weight. At the end of the dehydration assays (180 min), leaf discs were subjected to histochemical detection of H_2_O_2_ according to the previously described methodology [38]. For the *in vitro* drought stress tolerance assay, seven-day-old WT and transgenic (T_2_ generation) seedlings were removed from Murashige and Skoog (MS) germination medium for the WT and MS medium + 50 mg L^-1^ kanamycin (Sigma, St. Louis, MO, USA) for the transgenic lines and transferred to MS medium only (control treatment) or MS medium containing 15% polyethylene glycol 6000 (PEG-6000) (Merck, Darmstadt, Germany). The FW and root length of individual seedlings was measured 30 days after the treatments. At the end of the experiment, lipid peroxidation was determined by measuring the thiobarbituric acid reactive substance (TBARS) content according to the method described by [37]. For the soil dry-down experiment, 30-day-old WT and transgenic (T_1_ generation) plants, with an average of 10 to 15 leaves, were first transplanted into 20-liter pots. The pots contained Oxisol and washed sand as substrate in a ratio of 2:1. The plants were grown in greenhouse conditions for four weeks under temperature conditions of 25 ± 4°C and 80–90% humidity. The pots were then sealed with aluminum foil and the plants were subjected to water deficiency by the gradual suspension of the irrigation until they reached leaf predawn water potential of -1.5 MPa, or maintained leaf predawn water potential values of -0.2 to -0.4 MPa under control conditions, by means of daily irrigations. The leaf water potential was measured daily in the third completely expanded leaf from the apex of the plants, using a Scholander type pressure chamber (m670, Pms Instrument Co., Albany, USA). Each WT and transgenic line was represented by five biological replicates per treatment, obtained through micropropagation. The physiological analyzes, including the net CO_2_ assimilation rate (*A*), stomatal conductance to water vapor (*gs*) and the leaf transpiration rate (*E*) were performed in the morning (08:00–09:00 AM) using a portable photosynthesis system LI-6400 (Li-Cor) equipped with an artificial light resource (6400-02B RedBlue), as previously described [38].

### Identification and sequence analysis of citrus NF-Ys

We have queried the sweet orange genome data available at the Phytozome database (http://www.phytozome.org/citrus) with the 30 *Arabidopsis thaliana* NF-Y protein sequences [39] downloaded from TAIR (http://www.arabidopsis.org), using the TBLASTN tool. Statistically significant alignments were carefully inspected for the presence of the characteristic NF-Y motifs. We have carried out the identification of *Citrus clementina* NF-Ys by searching its genome data, also available at the Phytozome database (http://phytozome.jgi.doe.gov/pz/portal.html-!info?alias=Org_Cclementina), and using the same strategy as outlined for sweet orange.

Information about coding sequences (CDS), exon-intron structure, full-length sequences and predicted amino acid sequences of the sweet orange NF-Ys was obtained from the Phytozome database. The exon/intron gene structures were constructed using the Exon-Intron Graphic Maker (http://wormweb.org/exonintron). The physical locations of citrus NF-Ys were determined by confirming the starting position of all genes in each chromosome, using BLASTN searching against the local database of the *Citrus sinensis* Annotation Project (CAP; http://citrus.hzau.edu.cn/orange/). MapChart 2.30 software (https://www.wur.nl/en/show/Mapchart-2.30.htm) was used to plot the gene loci on the sweet orange chromosomes. GRAVY (grand average of hydropathy), molecular weight and isoelectric point (pI) of the deduced amino acid sequences were predicted with the PROTPARAM tool, available on the Expert Protein Analysis System (ExPASy) at the proteomics server (http://www.expasy.ch/tools/protparam.html). The subcellular localization was predicted using the WoLF PSORT tool (http://www.genscript.com/psort/wolf_psort.html). The alignments of predicted amino acid sequences were carried out with the ClustalX program [40] and shaded with BOXSHADE 3.21 software (http://www.ch.embnet.org/software/BOX_form.html). Phylogenetic trees were generated using the Neighbor-Joining method [41] with a bootstrap option of 1000 replications. The dendrograms were constructed by the MEGA6 program [42].

NF-Y proteins were searched against the Protein Data Bank (PDB; http://www.rcsb.org/pdb/) by BLASTP to identify the best template having similar sequence and known three-dimensional structure. The data were run into Phyre2 (Protein Homology/AnalogY Recognition Engine; http://www.sbg.bio.ic.ac.uk/phyre2) for predicting the protein homology modeling under the “intensive” mode. The protein structures of NF-Y were modeled at 90% confidence.

RNA-Seq data were downloaded from the CAP database [43] and used to generate the expression patterns of citrus NF-Ys in different tissues, namely callus, flower, leaf and fruit (flesh tissue). Stress-response expression was inferred from the transcript-per-million (TPM)-converted ratio of the count of *CsNF-Y* ESTs to the total number of clones present in non-normalized EST libraries (Assembly C52) produced from a range of tissues and stress treatments available in the HarvEST:Citrus 1.32 database (http://harvest-web.org/). The heat maps were generated using the Cluster 3.0 software.

### RNA extraction and quantitative real-time RT-PCR (qRT-PCR) analysis

All procedures of RNA extraction, cDNA synthesis and quantitative real-time RT-PCR (qRT-PCR) analysis were performed as previously described [44]. qRT-PCR primers were designed appropriately to avoid the conserved regions. The sequences of the primers used in the qRT-PCR reactions for *CsNF-YA5* were either 5'-CATTTCAGAATGGGGAAATCAT-3' and 5'-TTCTTCCTCATCTCACCCAAAG-3' for control and drought-stressed citrus samples, or 5'-TTGCAAGGGCTGGATACCTA-3 ' and 5'-CAACCCCTAGCCCTTCTCAT-3' for the *CsNF-YA5* overexpressing transgenic tobacco samples. B-actin (*CsACT*) or GAPDH (*NtGAPDH*) genes were amplified together with the target gene (*CsNF-YA5*) as endogenous controls to normalize expression between the different tissue samples of citrus or tobacco, respectively. The primers CsACT-F (5'-TTAACCCCAAGGCCAACAGA-3'), CsACT-R (5'-TCCCTCATAGATTGGTACAGTATGGAC-3'), NtGAPDHRT-F (5'-TCAAACCCTTCCACCAACTC-3') and NtGAPDHRT (5’-CTAATCGCCCAATTCTTCCA-3') were used in the qRT-PCR reactions. The expression values of the genes were calculated by means of the 2^-ΔΔCt^ method [45]. Confirmation of the amplification of specific products was performed by analyzing the dissociation curves with the Mx3005P qPCR analysis software. Data were obtained from a pool of three biological replicates that were individually validated.

### Cloning of *CsNF-YA5* and generation of transgenic tobacco plants

The complete coding sequence of *CsNF-YA5* was obtained from its amplification in cDNA samples of roots of Rangpur lime (*C*. *limonia* Osb.) subjected to water deficit, using the primers 5'-CAAGATGGGATTAATGGACAAGAAC-3 ' and 5'-CCCTCCAACGTCAAGGAACT-3'. The amplified cDNA fragment was inserted into the pGEM-T vector (Promega, USA) by means of the TA cloning system and used in transformation of *Escherichia coli*, strain TOP 10. Subsequently, the amplified fragment was taken from pGEM-T by means of digestion with the restriction enzyme *Not*I, and subcloned in sense orientation at the same restriction site of the plasmid pUC118/CaMV 35S, which contains the promoter and terminator sequences of the 35S gene of cauliflower mosaic virus (CaMV 35S). The expression cassette was then excised from pUC118/CaMV 35S, by means of digestion with *HindIII* and *SalI*, and inserted into the same restriction sites of pCAMBIA 2301 vector (Cambia, Australia). This vector contains the reporter gene *uidA* and the selective *nptII* gene under the control of the constitutive promoter CaMV 35S. The recombinant vector was then introduced into the *Agrobacterium tumefaciens* strain of EHA-105 and used in genetic transformation experiments of *N*. *tabacum*.

The experiments of genetic transformation of tobacco were carried out using the methodology described by Cidade et al. [46]. In brief, leaf segments of ~1 cm^2^ were excised and placed in contact with bacterial suspension (OD_600_ = 0.5), for 15 minutes at room temperature and under gentle shaking. The excised explants were subjected to (i) bacterial suspension coculture containing the pCAMBIA 2301 vector with the *CsNF-YA5* gene, (ii) bacterial suspension culture containing pCAMBIA 2301 without the insertion of the gene of interest and (iii) non-cocultivated explants with *Agrobacterium* as control of the experiment. After the development of the aerial parts, the plants were multiplicated by micropropagation, in which the aerial parts were excised with the use of sterile forceps and scalpel and inoculated in MS medium plus 300 mg mL^-1^ timentin. At least five biological replicates (clones) were obtained for each WT and transgenic lines with empty vector (PC) or overexpressing *CsNF-YA5* (NF12, NF15, NF16, NF20 and NF22). The plants were submitted to the acclimation phase, where they remained for one week in distilled and autoclaved water, and covered with plastic bags with cavity at the ends. Afterwards, the plants were transferred to plastic vessels with a capacity of 5-L, containing autoclaved soil substrate and sand in a ratio of 2:1, where they remained for three weeks in a growth room with controlled temperature and luminosity. Screening by PCR and histochemical GUS assays were used to identify the transgenic plants, according to the methodologies described, respectively, by Cidade et al. [46] and Jefferson et al. [47].

### Statistical analysis

The statistical analysis was carried out with the SISVAR and BioEstat softwares which tested the experiments as a completely randomized design. Statistical differences were assessed based on the analysis of variance (ANOVA) and averages were separated by means of the Student’s *t*-test, with a critical value of *P*≤ 0.05, *P*≤ 0.01 and *P*≤ 0.001. The leaf-disc dehydration tolerance assay was composed by three plant replicates per treatment, with five leaf discs per replication. Regression lines were fitted by the method of least squares and the significance of the coefficient of determination (r2) was verified by the F-test. The *in vitro* drought stress tolerance assay contained five replicate plates per treatment composed of fifteen seedlings for each line. The soil dry-down experiment consisted of five plants per treatment, with each plant considered as an experimental unit.

## Results

### The citrus *NF-Y* gene family

BLAST searches using the 30 amino acid sequences of the complete set of *A*. *thaliana* NF-Y proteins as query sequences resulted in the identification of a total of 22 different *Citrus* species *NF-Y* encoding protein genes (*CsNF-Y*s) in the sweet orange genome, including six *CsNF-YA*s, 11 *CsNF-YB*s and five *CsNF-YC*s (Table 1). BLAST searches against the genome sequence of Clementine mandarin using the same strategy as outlined for sweet oranges revealed that both *Citrus* species contain exactly the same number of *NF-Y* genes (Table 1). Phylogenetic analysis showed that there is a *NF-Y* ortholog in the genome of Clementine mandarin for each sweet orange *NF-Y* gene, while two *CsNF-YA*s (*CsNF-YA5* and *CsNF-YA6*), four *CsNF-YB*s (*CsNF-YB1*, *CsNF-YB2*, *CsNF-YB5* and *CsNF-YB11*) and one *CsNF-YC* (*CsNF-YC4*) have no close homologs in *A*. *thaliana* (S1–S3 Figs and Table 1).

**Table 1:** Citrus *NF-Y* transcription factor encoded genes.

Name	*Locus* (Phytozome)	*Locus* (CAP)	*C*. *clementina* ortholog	*A*. *thaliana* ortholog	Chromosome location	Polypeptide length (MW)	pI	GRAVY	Predicted subcellular location
CsNF-YA1	orange1.1g019036m	Cs2g30350	Ciclev10015808m	NF-YA1 NF-YA9	chr2: 29,712,980..29,717,207	347 (37.2kDa)	8.26	-0.964	nucleus
CsNF-YA2	orange1.1g021081m	Cs6g13560	Ciclev10012232m	NF-YA2 NF-YA10	chr6: 14,987,576..14,992,571	317 (34.6kDa)	9.27	-0.597	nucleus
CsNF-YA3	orange1.1g019782m	Cs1g17780	Ciclev10026020m	NF-YA3 NF-YA8 NF-YA5 NF-YA6	chr1: 20,916,460..20,920,299	336 (36.7kDa)	8.67	-0.617	nucleus
CsNF-YA4	orange1.1g026474m	Cs2g09780	Ciclev10016491m	NF-YA4 NF-YA7	chr2: 7,234,224..7,238,903	238 (26.5kDa)	9.15	-0.733	extracellu-lar
CsNF-YA5	orange1.1g019764m	Cs7g01720	Ciclev10032207m	-	chr7: 461,288..464,375	336 (36.8kDa)	9.54	-0.749	nucleus
CsNF-YA6	orange1.1g017825m	Cs9g12370	Ciclev10005144m	-	chr9: 10,757,085..10,763,538	365 (39.9kDa)	9.44	-0.723	nucleus
CsNF-YB1	orange1.1g045289m	orange1.1t03346	Ciclev10027508m	-	chrUn: 51,410,005..51,410,973	71 (8.0kDa)	4.79	-0.58	chloroplast
CsNF-YB2	orange1.1g027605m	orange1.1t03346	Ciclev10027508m	-	chrUn: 51,410,005..51,410,973	221 (24.1kDa)	6.33	-0.725	nucleus
CsNF-YB3	orange1.1g036580m	Cs4g08780	Ciclev10009623m	NF-YB3 NF-YB2	chr4: 5,787,274..5,788,494	186 (20.2kDa)	6.53	-0.788	nucleus
CsNF-YB4	orange1.1g045194m	-	Ciclev10003157m	NF-YB4	-	157 (17.6kDa)	8.37	-0.947	nucleus
CsNF-YB5	orange1.1g044287m	Cs9g04610	Ciclev10006769m	-	chr9: 2,565,158..2,565,691	188 (20.9kDa)	5.43	-0.84	nucleus
CsNF-YB6	orange1.1g026469m	Cs2g01680	Ciclev10016498m	NF-YB6	chr2: 563,303..566,657	238 (26.5kDa)	6.72	-0.668	mitochon-drion
CsNF-YB7	orange1.1g047516m	Cs1g09850	Ciclev10003558m	NF-YB7	chr1: 11,604,325..11,604,969	214 (24.1kDa)	5.80	-1.014	nucleus
CsNF-YB8	orange1.1g030647m	Cs6g04240	Ciclev10012920m	NF-YB8 NF-YB10	chr6: 4,871,183..4,873,746	174 (19.0kDa)	5.67	-0.833	nucleus
CsNF-YB9	orange1.1g038325m	Cs4g08720	Ciclev10010391m	NF-YB9	chr4: 5,742,982..5,745,273	231 (25.4kDa)	6.05	-0.542	nucleus
CsNF-YB10	orange1.1g030547m	Cs7g27760	Ciclev10032893m	NF-YB8 NF-YB10	chr7: 28,445,326..28,456,064	175 (18.9kDa)	5.81	-0.685	nucleus
CsNF-YB11	orange1.1g038014m	Cs5g32730	Ciclev10024294m	-	chr5: 33,945,582..33,946,004	140 (15.6kDa)	5.62	-0.805	nucleus
CsNF-YC1	orange1.1g026901m	orange1.1t02576	Ciclev10032646m	NF-YC1 NF-YC4	chrUn: 39,384,571..39,387,608	231 (25.0kDa)	5.05	-0.455	nucleus
CsNF-YC2	orange1.1g024265m	orange1.1t03257	Ciclev10007003m	NF-YC2	chrUn: 50,081,198..50,085,450	270 (30.2kDa)	5.81	-0.644	nucleus
CsNF-YC3	orange1.1g019814m	Cs3g13490	Ciclev10002193m	NF-YC3 NF-YC9	chr3: 17,935,028..17,937,988	335 (37.3kDa)	9.24	-0.641	nucleus
CsNF-YC4	orange1.1g047870m	Cs5g03650	Ciclev10023545m	-	chr5: 1,967,345..1,967,909	102 (11.2kDa)	9.61	-0.034	chloroplast
CsNF-YC5	orange1.1g045847m	Cs6g18220	Ciclev10013435m	NF-YC12	chr6: 18,422,446..18,425,136	237 (27.1kDa)	5.24	-0.526	nucleus

Most *CsNF-Y* genes were precisely mapped on the sweet orange chromosomes by homology searches against the sweet orange genome assembly available at the CAP database. Some *CsNF-Y*s could not be located on any chromosome (ChrUN) because of an incomplete physical map for sweet orange. *CsNF-Y* loci were distributed throughout all the sweet orange chromosomes, except for the chromosome 8, at densities varying from one to three genes per chromosome (S4 Fig and Table 1). Two pairs of paralogous *CsNF-Y* genes were detected based on both phylogenetic analysis and chromosomal location. One of these pairs, *CsNF-YB8/10* (82.2% similarity), was putative segmental duplicates according to criteria of Gu et al. [48]: the length of aligned sequence covers >80% of the longer gene and the similarity of the aligned region is >70%. The other gene pair, *CsNF-YB3/9* (~40 kb proximity to each other), was putative tandem duplicates according to the criteria of Hanada et al. [49]: tandem duplicates are genes in any gene pair that belong to the same gene family and are located within 100 kb each other.

Analysis of the exon-intron structure of all *CsNF-Y* genes was carried out using the sweet orange gene models annotated in Phytozome (S5 Fig). In general, there were similarities in the number and length of the exons, but not of the introns, among *CsNF-Y*s and their putative orthologs from *Arabidopsis* (*AtNF-Y*s). All *CsNF-YA* genes were interrupted by three to four introns in their coding regions. Besides, most *CsNF-YA* genes contained introns embedded in their 5’- and/or 3’-UTR. No intron was present in 6 members from the *CsNF-YB* family, while the other members showed variations from 1 to 5 introns in their coding regions. Two *CsNF-YB* members, *CsNF-YB8/10*, contained introns in their 5’-UTR. There were 3 *CsNF-YC* members (*CsNF-YC1/2/4*) containing no or only one intron, while the others contained 2 or 6 introns in their coding regions. Two *CsNF-YC* members, *CsNF-YC*2/3, also contained introns in their 5’- and/or 3’-UTR. Interestingly, the pair of paralogous genes showed some variations in their exon-intron structures, such as different number of introns (*CsNF-YB8/10*) and length of exons (*CsNF-YB3/9*). These data suggest that the organization of *CsNF-Y* genes is dynamic and rapidly evolving.

The *CsNF-Y* genes encode proteins ranging from 71 (8 kDa) to 365 (39.9 kDa) amino acids in length, and pI values ranging from 4.79 to 9.54 (Table 1). The average protein length of CsNF-YAs, CsNF-YBs and CsNF-YCs were 323 (35.3 kDa), 176.5 (19.5 kDa) and 202.2 (22.5 kDa) amino acids respectively, while their average pI were, respectively, 9.05, 6.01 and 7.07. All the CsNF-Y proteins had a negative GRAVY score, indicating that they are hydrophilic proteins (Table 1). Most CsNF-Ys were predicted to be located in the nucleus, with some of them also targeted to chloroplast and mitochondrion (Table 1).

**Fig 1:**
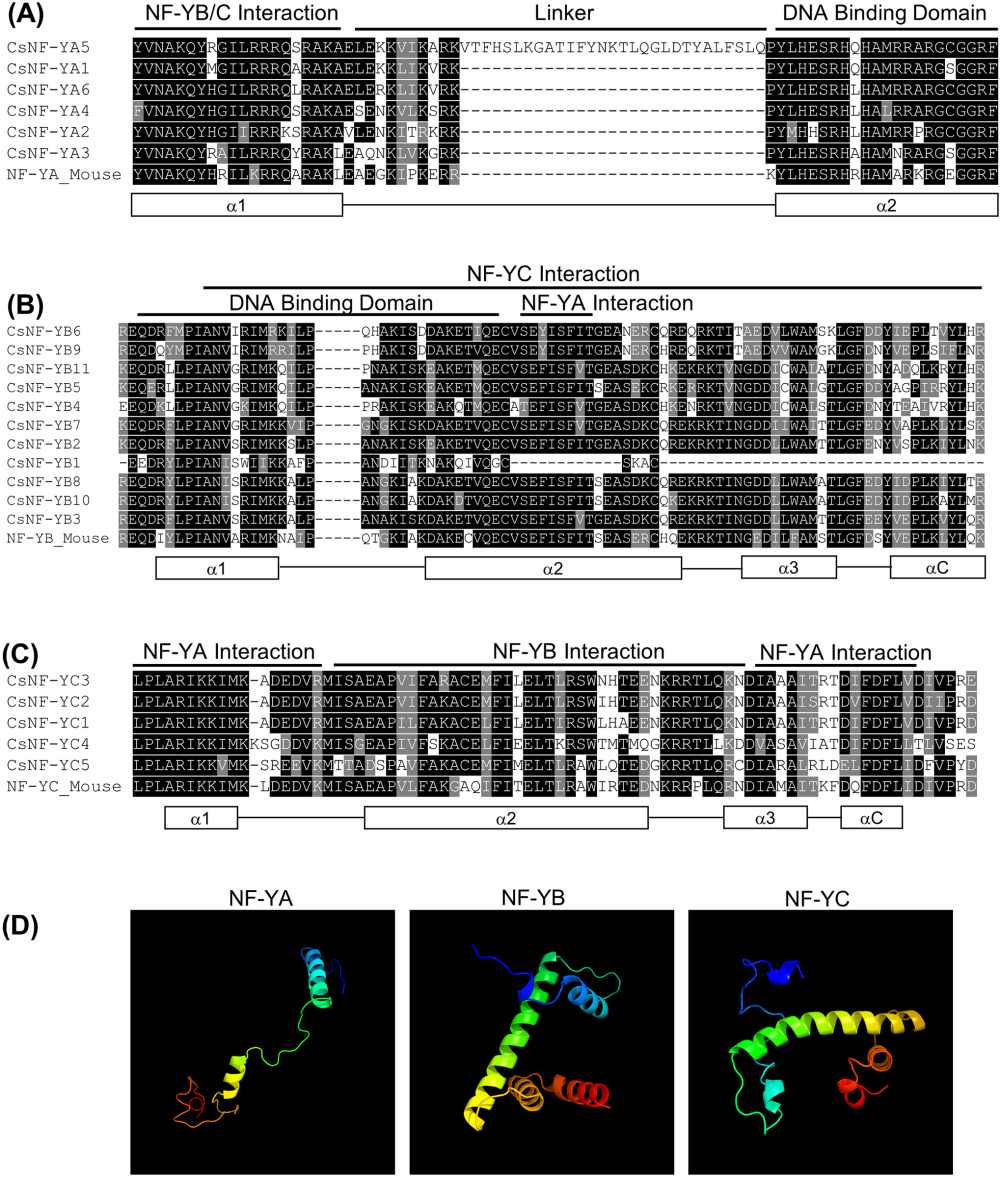
Multiple alignments and predicted structure of conserved regions of CsNF-Y family members. Sequence alignments among the highly conserved domains of NF-YA (**A**), NF-YB (**B**) and NF-YC (**C**) proteins of *C*. *sinensis* (Cs) and *Mus musculus* (Mouse). The DNA binding domain and the domains required for interaction with the other subunits previously defined in yeast and mammals are indicated. The secondary structures, alpha-helices (rectangles) and coils (black lines), are represented on the bottom of the alignment, based on Romier et al. [55]. Predicted structures of NF-Y conserved regions using the protein homology modeling of Phyr2 **(D)**. Images of the models were coloured in a rainbow from blue to red from the N-terminus to the C-terminus.

Multiple alignments showed that CsNF-Y proteins contain the evolutionarily conserved domains that are responsible for heterodimerization, heterotrimerization and DNA interactions at the CCAAT sites (Fig 1). The conserved core region of CsNF-YA is ~53 amino acids in length and composed of two sub-domains, one mediating the NF-YB/C interaction and the other responsible for CCAAT binding (Fig 1A). These sub-domains are separated from each other by a relatively conserved linker. The CsNF-YB subunit core region is ~88 amino acids in length and contains the NFYC interaction domain that extends across two independent regions and partly overlaps with the DNA-binding and NF-YA interaction domains (Fig 1B). This region has structural similarities with the histone-fold motif (HFM) of the core histone H2B [50]. The CsNF-YC subunit core region is ~79 amino acids long and possesses the highly conserved domains for NF-YA and NF-YB interactions, with the former extending across two separate regions (Fig 1C). This region is also characterized by an HFM, but that is more similar to the core histone H2A than H2B [50]. The secondary structures of the conserved core regions of NF-Y proteins were predicted to be composed of α-helices and coils, using the protein homology modeling of Phyr2 (Fig 1D). The NF-YA conserved region was comprised of two α-helices, one in each sub-domain. The NF-YB and NF-YC conserved regions were comprised of four α-helices that are located within all the functionally important domains for NF-Y interaction and DNA binding.

### Expression analysis of *CsNF-Y* genes in different tissues and in response to abiotic and biotic stresses

**Fig 2:**
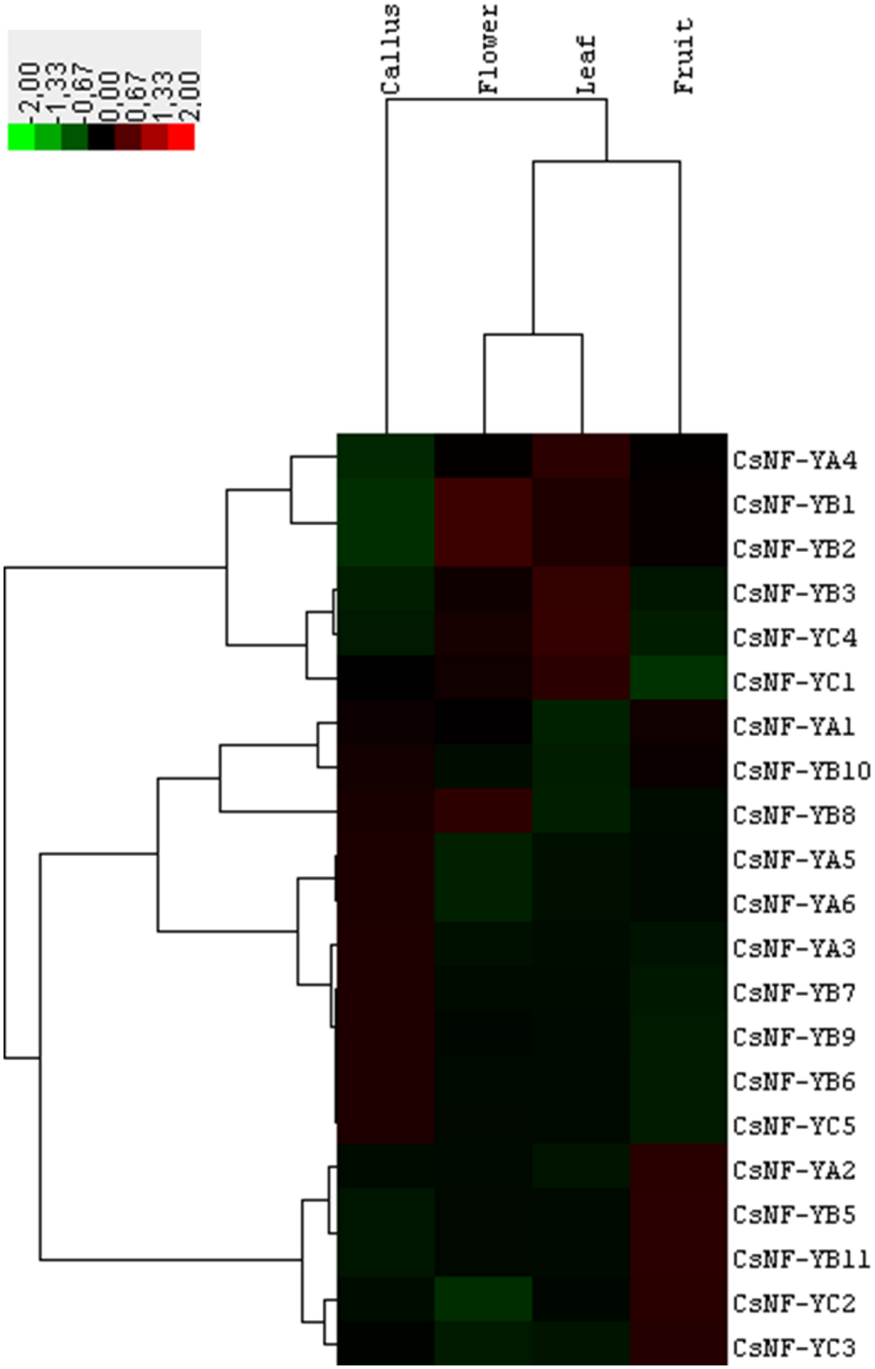
Heatmap of expression of *CsNF-Y*s in different tissues of sweet orange. The color scale shown represents RPKM-normalized log_2_-transformed counts.

**Fig 3:**
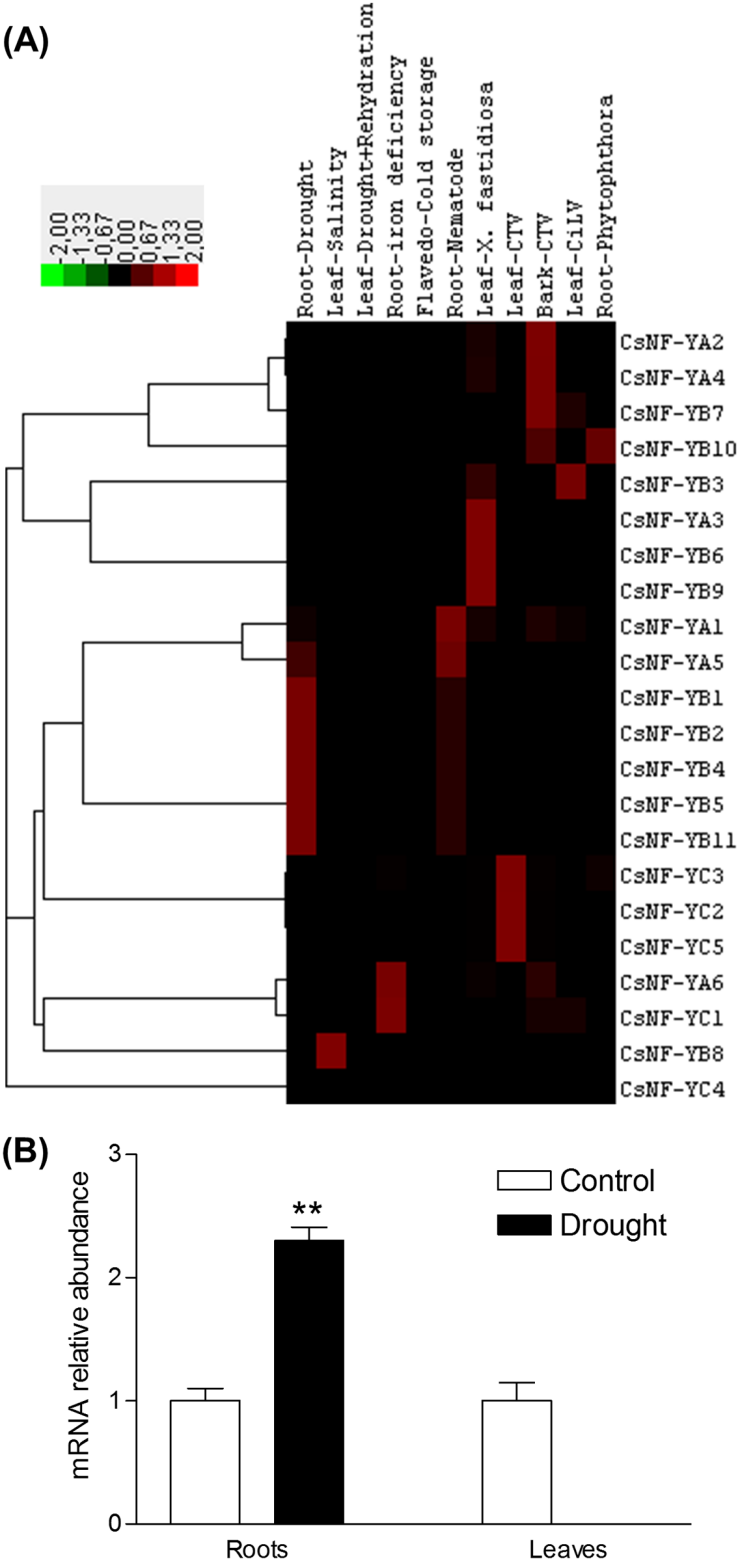
Expression analysis of *CsNF-Ys*. Heatmap of expression of *CsNF-Ys* in response to different stress treatments **(A)**. The color scale shown represents TPM-normalized log_2_-transformed counts. qRT-PCR expression analysis of *CsNF-YA5* **(B)**. *CsNF-YA5* mRNA abundance in leaves and roots of sweet orange plants grafted on Rangpur lime, subjected to control (irrigated) and drought treatments. The data are means ± SE of three biological replicates in which *β-actin* transcripts were used as internal controls.**Significantly different from control treatment at *P* ≤ 0.01.

Expression analysis from RNA-seq data showed that, except for *CsNF-YB4*, all the *CsNF-Y* genes are preferentially expressed in one of the major sweet orange tissues (Fig 2). We have observed four main clusters of expression. Cluster 1 corresponded to *CsNF-Y* genes highly expressed in callus, while those from cluster 2 included *CsNF-Y*s with high expression in fruit. Cluster 3 and Cluster 4 included highly expressed *CsNF-Y* genes in leaf and flower, respectively. Expression analysis from EST data demonstrated that, except for *CsNF-YC4*, all the *CsNF-Y* genes were also expressed in response to one or more stress treatments (Fig 3A). Major clusters of expression responses were associated with the drought, nematode, Citrus tristeza virus (CTV) and *X*. *fastidiosa* treatments. Drought induced the expression of one *CsNF-YA* (*CsNF-YA5*) and five *CsNF-YB*s (*CsNF-YB1/2/4/5/11*) in roots. All these genes, plus *CsNF-YA1*, were also expressed in roots in response to nematode treatment. CTV induced the expression of four *CsNF-YA*s (*CsNF-YA1/2/4/6*), two *CsNF-YB*s (*CsNF-YB7/10*) and one *CsNF-YC* (*CsNF-YC1*) in barks, and three *CsNF-YC*s (*CsNF-YC2/3/5*) in leaves. *X*. *fastidiosa* induced the expression of one *CsNF-YA* (*CsNF-YA3*) and three *CsNF-YB*s (*CsNF-YB3/6/9*) in leaves. Taken together, these data indicated that *CsNF-Y*s exhibit a tissue-specific and abiotic and/or biotic stress-inducible expression pattern.

### *CsNF-YA5* expression analysis in response to drought stress

Our comprehensive analysis has provided a good starting point for the functional characterization of citrus *NF-Y*s. It helped us to choose *CsNF-YA5* as the first citrus *NF-Y* family member to be functionally characterized because it was the only *CsNF-YA* gene to be induced by drought treatment. Besides, *CsNF-YA5* has no close homologs in *Arabidopsis*, representing therefore a novel *NF-YA* gene. It shares the highest amino acid sequence similarity with the predicted nuclear transcription factor Y subunit A-4 from *Theobroma cacao* (XP_007052521.2; 65% similarity). qRT-PCR expression analysis revealed distinct expression patterns of *CsNF-YA5* in leaves and roots of drought-stressed citrus plants (Fig 3B). A significant induction of the expression of *CsNF-YA5* (2.5-×) was observed in roots of plants subjected to drought treatment, when compared to that of the control treatment. On the other hand, the expression of *CsNF-YA5* was completely repressed in leaves of plants exposed to drought treatment.

### *CsNF-YA5* overexpression in transgenic tobacco plants

The complete coding sequence of *CsNF-YA5* was amplified from root cDNA samples of Rangpur lime (S6 Fig), inserted into pCAMBIA 2301 under the control of the constitutive promoter CaMV 35S and used in the *Agrobacterium-*mediated genetic transformation of tobacco. Several shoots representing distinct transformation events were obtained from the genetic transformation experiments (S7 Fig). Five independent transgenic lines were analyzed by PCR, all of which showed amplification of a fragment compatible with 800-bp of *nptII* gene (S8A Fig). Histochemical staining analysis of GUS was also used to confirm the transgenic nature of the plants (S8B Fig). The expression profile of *CsNF-YA5* in mature leaves of WT and transgenic lines was examined by qRT-PCR analysis (S8C Fig). All the analyzed *CsNF-YA5*-overexpressing transgenic lines presented higher levels (2.5–4.0-×) of *CsNF-YA5* expression when compared to the WT and empty vector (PC) control lines.

### Dehydration tolerance assay of *CsNF-YA5*-overexpressing tobacco plants

**Fig 4:**
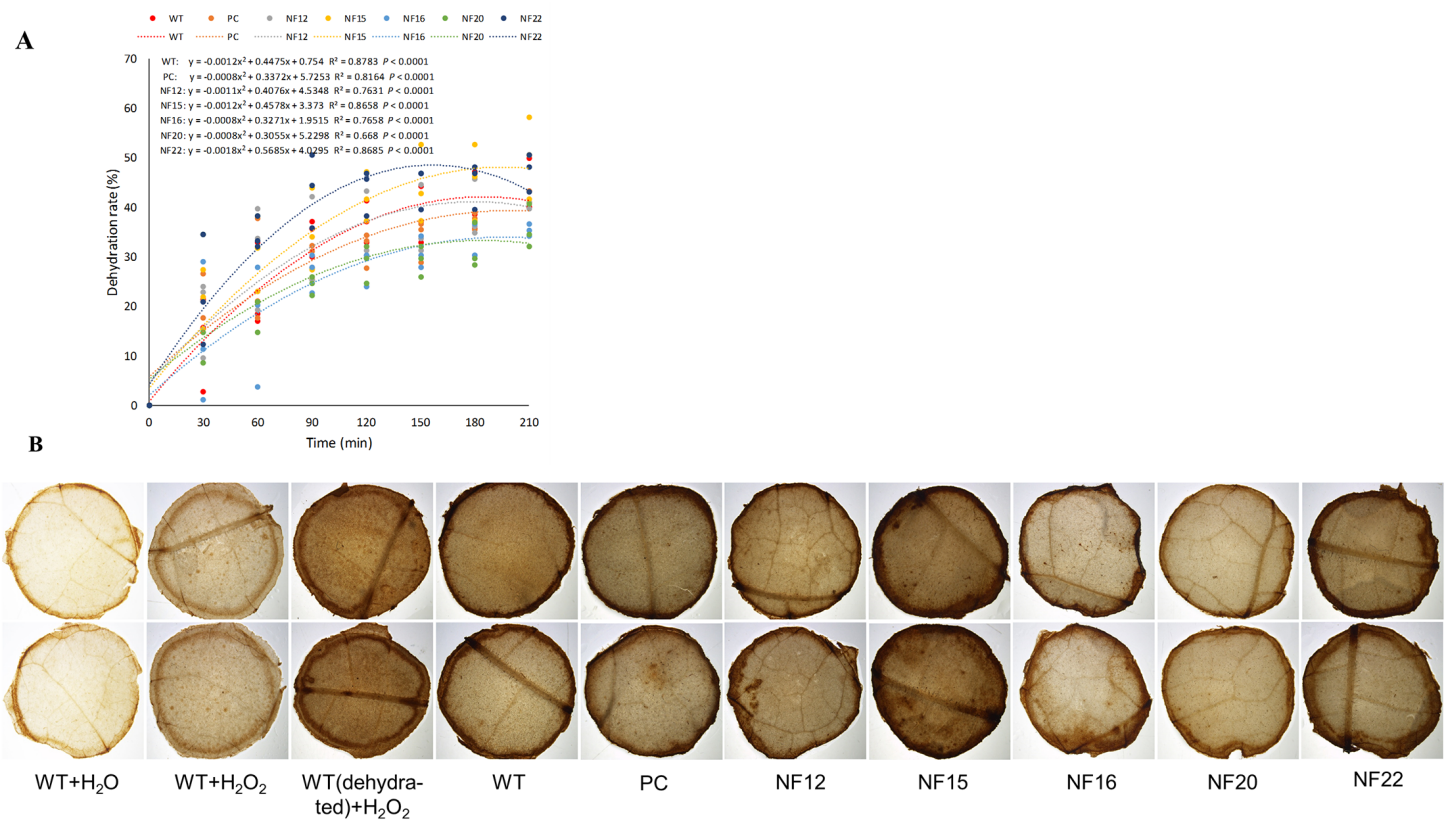
Leaf-disc dehydration tolerance assay. Dehydration rate in leaf discs of control (NT and PC) and *CsNF-YA5*-overexpressing transgenic (NF12, NF15, NF16, NF20, NF22) lines for 210 min at room temperature **(A)**. Measurement by fresh weight reduction were carried out every 30 min. Regression lines were fitted to the data using least-squares regression analysis. Each point represents the mean of three plant replicates per treatment, with five leaf discs per replication. *In situ* detection of H_2_O_2_ in tobacco leaf discs treated with DAB **(B)**. Representative photographs showing staining of H_2_O_2_ in leaf disks of WT and transgenic lines with empty vector (PC) or overexpressing *CsNF-YA5* (NF12, NF15, NF16, NF20, NF22) after 180 min dehydration. WT leaf discs before dehydration (WT+H_2_O_2_) and treated with H_2_O_2_ [WT(dehydrated)+H_2_O_2_] or only water (WT+H_2_O), after 180 min dehydration, were used as controls.

Leaf discs of control (WT and PC) and *CsNF-YA5*-overexpressing transgenic (NF12, NF15, NF16, NF20, NF22) lines were subjected to dehydration stress for 210 min. The time course of water loss rate showed that the leaf discs of NF16 and NF20 dehydrated slower, NF15 and NF22 faster, and NF12 similarly than those of the WT and PC control plants (Fig 4A).

H_2_O_2_ production was analyzed in leaf discs of tobacco plants subjected to 180 min of dehydration using the DAB-HCL histochemical staining analysis. According to the results, four (NF12, NF16, NF20 and NF22) out of the five tested *CsNF-YA5*-overexpressing transgenic lines showed significantly clearer staining than those of the control plants (WT and PC), indicating a lower H_2_O_2_ production (Fig 4B). NF15 presented a more intense coloration and similar to that of the control plants. H_2_O_2_ production was lower in non-dehydrated and H_2_O_2_-treated WT than in the WT subjected to dehydration and treated with H_2_O_2_. We have not observed, as expected, darkening of the leaf discs of WT treated with water instead of DAB, when compared to the leaf discs immersed in DAB solution (Fig 4B).

### *In vitro* drought stress tolerance assay of *CsNF-YA5*-overexpressing tobacco plants

**Fig 5:**
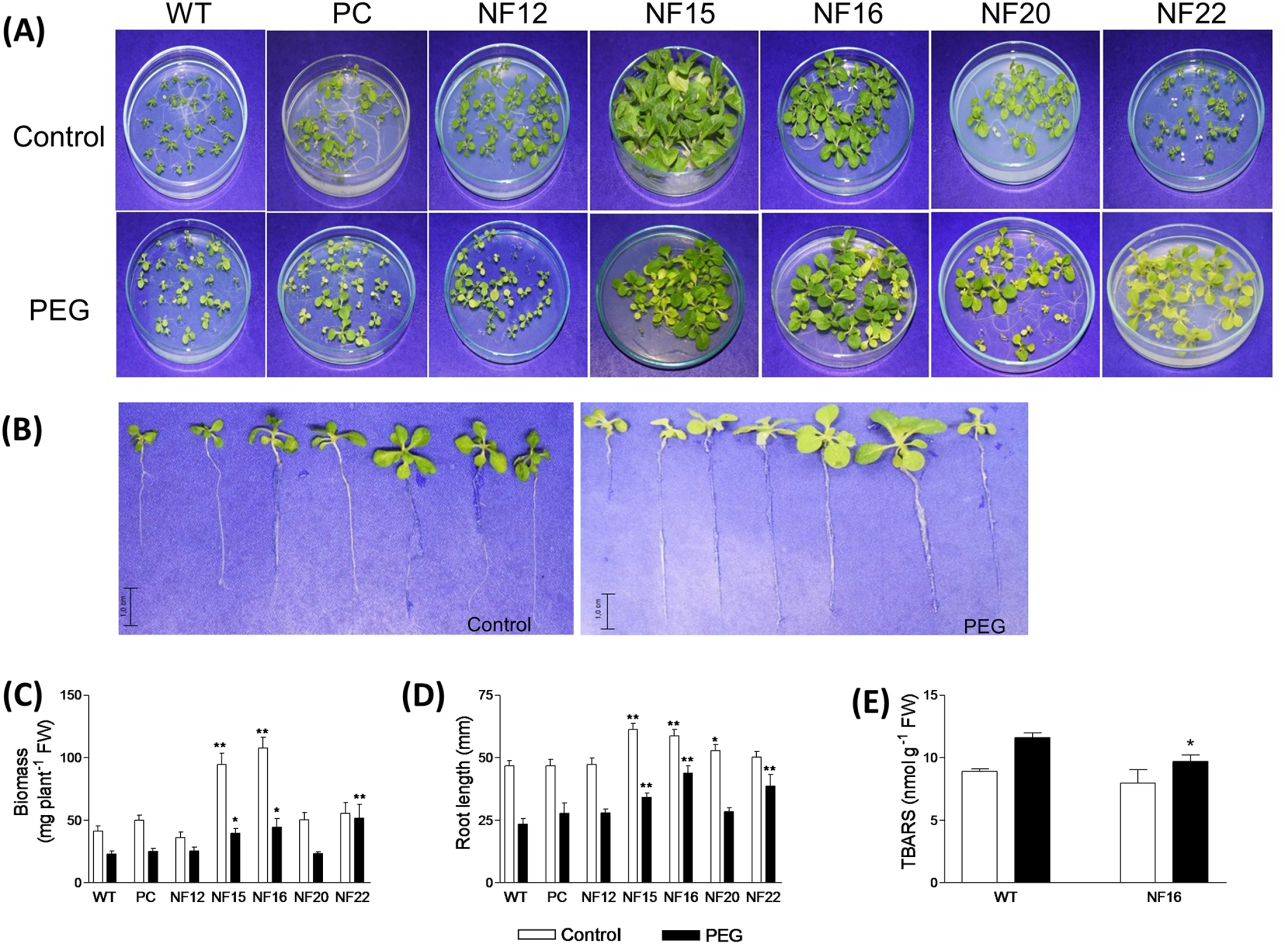
*In vitro* drought stress tolerance assay of *CsNFYA5*-overexpressing transgenic lines. Representative phenotypes of control (WT and PC) and *CsNFYA5* transgenic (NF12, NF15, NF16, NF20 and NF22) lines grown under control and PEG treatments for 30 days **(A, B)**. Shown from left to right in panel B: WT, PC, NF12, NF15, NF16, NF20 and NF22. Seedling biomass and root length, respectively, of control and transgenic lines under control and PEG treatments for 30 days **(C, D)**. The data are means ± SE of five technical replicates composed of fifteen seedlings for each line. Total thiobarbituric acid reactive substances (TBARS) concentration in seedlings of WT and transgenic line NF16 under control and PEG treatments for 30 days **(E)**. The data are means ± SE of six technical replicates composed of fifteen seedlings for each line. Statistically significant differences at *P* ≤ 0.01 (**) or *P* ≤ 0.05 (*) between control (WT) and *CsNFYA5* transgenic lines, at the respective treatment, are indicated.

In order to evaluate the effects of the *CsNF-YA5* overexpression on drought stress tolerance of the transgenic lines at the level of whole plant, control and transgenic plants were subjected to the *in vitro* drought stress tolerance assay. The representative phenotypes of WT and transgenic plants under control and drought treatments are shown in Fig 5A and 5B. In general, seedlings from the transgenic lines tended to have higher biomass (Fig 5C) and root length (Fig 5D) than the WT under both control and PEG treatment. Analysis of the TBARS content showed that the transgenic line NF16 had a lower index of lipid peroxidation than the WT under PEG treatment (Fig 5E).

### Physiological analysis of *CsNF-YA5*-overexpressing tobacco plants

**Fig 6:**
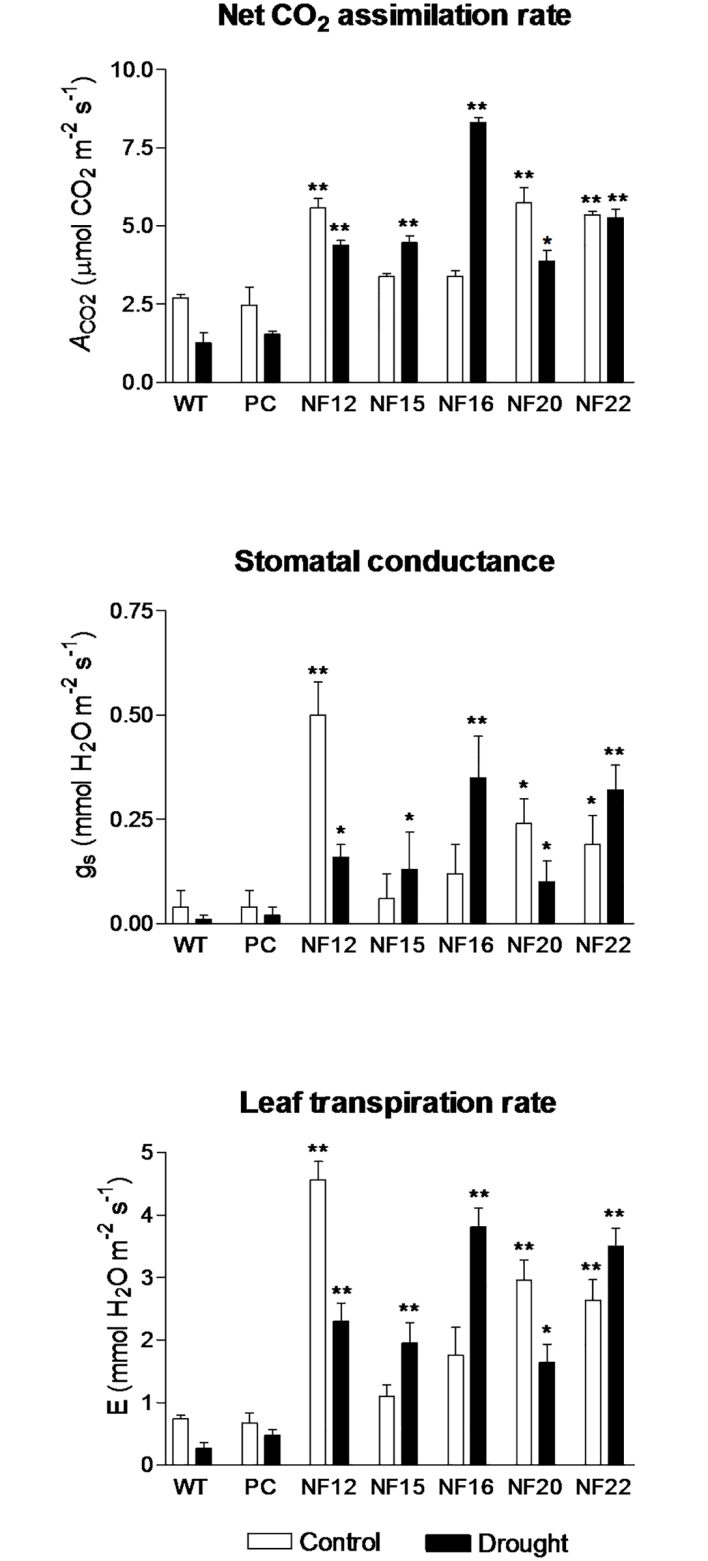
Physiological analysis of *CsNFYA5*-overexpressing transgenic lines. Control (WT and PC) and *CsNFYA5* transgenic (NF12, NF15, NF16, NF20 and NF22) lines were subjected to control (irrigated) and drought treatments under greenhouse conditions. The data are means ± SE of five plant replicates per treatment for each line. Statistically significant differences at *P* ≤ 0.01 (**) or *P* ≤ 0.05 (*) between control (WT) and *CsNFYA5* transgenic lines, at the respective water treatment, are indicated.

To understand how the water deficit influences the responses of the *CsNF-YA5*-overexpressing transgenic lines at the physiological level, control and transgenic lines were subjected to the soil dry-down experiment under greenhouse conditions. Analysis of the net assimilation rate of CO_2_ (*A*), stomatal conductance to water vapor (*gs*) and the leaf transpiration rate (*E*) was carried out in plants under control (irrigated) and drought conditions. All the *CsNF-YA5*-overexpressing transgenic lines showed values of *A*, *gs* and *E* significantly higher than the control lines (WT and PC) in both irrigated and drought treatments (Fig 6). Another interesting observation was that, in contrast to the control lines, three (NF15, NF16 and NF22) out of the five *CsNF-YA5*-overexpressing transgenic lines exhibited higher rates of *A*, *gs* and *E* in the drought than in the irrigated treatment.

## Discussion

The number of genes identified for each citrus NF-Y subunit (Table 1) is lower than that reported for *A*. *thaliana* [39]. In light of this, the presence of six CsNF-YAs, 11 CsNF-YBs and five CsNF-YCs in citrus could result in the formation of only 330 unique trimetric complexes, a relatively small number when compared to 1,000 unique complexes that could be theoretically combined in *A*. *thaliana* (10 NF-YA, 10 NF-YB and 10 NF-YC subunits). However, the number of potential unique NF-Y complexes has been shown to be much smaller than previously anticipated, due to the specificity of expression and interactions among the different NF-Y subunits [1]. The lower number of NF-Y genes found in the citrus genome may be accounted for by the fact that *A*. *thaliana* has undergone two additional rounds of recent whole-genome duplication (WGD) when compared to citrus [35]. By the other hand, the lack of close homologs in *A*. *thaliana* for some citrus NF-Ys (S1–S3 Figs) suggests that more recent gene duplication events have contributed to the expansion of the family in citrus. The results of phylogenetic analyses (S1–S3 Figs) and chromosome mapping (S4 Fig) suggest that both segmental (e.g. *CsNF-YB8* and *CsNF-YB10*) and tandem (e.g. *CsNF-YB3* and *CsNF-YB9*) duplications have contributed to the *CsNF-Y* gene expansion in the citrus genome.

Gene-structure analysis of the *CsNF-Y* gene family revealed that *CsNF-YA*s have a more complex exon-intron organization than most *CsNF-YB*s and *CsNF-YC*s (S5 Fig), consistently with the previously reported organization of *NF-Y* genes in *Arabidopsis* and *Brassica napus* [51], *Phaseolus vulgaris* [52] and *Solanum lycopersicum* [53]. Notably, most *CsNF-YA*s and some *CsNF-YB*s and *CsNF-YC*s contained one intron in their 5’- and/or 3’-UTR. In *Medicago trunculata*, retention of the 5’-UTR intron in alternative spliced variants of *NF-YA1* leads to the synthesis of a small peptide that destabilizes the mRNA [54]. In *M*. *trunculata*, *A*. *thaliana* and *Oryza sativa*, most *NF-YA*s have at least one intron in the 5’-UTR [1], suggesting that this post-transcriptional regulatory mechanism might be conserved among plant NF-YA genes. *NF-YA* transcripts are also regulated at post-transcriptional level by the action of microRNA169 (miR169) that binds to the 3’-UTR and promotes their cleavage by the slicing protein Argonaute 1 [4,8].

Multiple alignments showed that CsNF-Y proteins have the conserved regions involved in protein interaction and DNA binding (Fig 1), which are highly similar from mammals (such as mouse) to plants [50,55]. This high degree of conservation strongly suggests the maintenance of ancestral functions related to the complex formation. From secondary structure prediction analysis, the NF-YA conserved region was divided into an α-helical N-terminal region involved in NF-YB/C interactions and a small coiled C-terminal region involved in DNA binding [55]. For DNA binding, the α1 and α2 of NF-YB and the α1 of NF-YC are also required [50]. The NF-YB helix α2 and NF-YC helices α1 and αC were shown to influence NF-YA binding [50,55]. The NF-YB and NF-YC α3s have been implicated in interactions with TBP, the TATA box-binding subunit of the general initiation factor TFIID [50]. The NF-YC αC region is a target element for gene-specific regulatory proteins, such as SP1, SREBP1, RF-X and C/EBP [55].

Orthology prediction is an important tool for transferring functional information between two species. *CsNF-YA1* is orthologous to *AtNF-YA1* and *AtNF-YA9* (S1 Fig and Table 1) which have been involved, respectively, in flowering time control [15] and fertility [56]. *CsNF-YA2* is orthologous to *AtNF-YA2* and *AtNF-YA10* that have been implicated in the control of root growth and architecture [57]. *CsNF-YA3* is orthologous to the *AtNF-YA3*, which is involved in nitrogen nutrition [58]. *CsNF-YA4* is orthologous to the *AtNF-YA4* that has been shown to participate in the ER-stress and unfolded protein response (UPR) [3]. The known *Arabidopsis* regulators of photoperiod dependent flowering time, NF-YB2/3 and NF-YC3/9 [15–18,24], have highly significant orthology matches to CsNF-YB3 and CsNF-YC3, respectively (S2 and S3 Figs and Table 1). Therefore, it is expected that most of these conserved citrus NF-Ys subunits are involved in similar developmental processes and stress responses as reported in *A*. *thaliana*.

Expression analysis from RNA-seq data revealed that *CsNF-Y*s exhibit a tissue-specific expression pattern (Fig 2), as previously reported for NF-Ys of *A*. *thaliana* [39], *Brachypodium distachyon* [59], *B*. *napus* [51], *Setaria italica* [60], *Glycine max* [61], *P*. *vulgaris* [52] and *S*. *lycopersicum* [53]. Such specific expression profiles indicate a sub-functionalization of the subunit members and provide certain clues about the potential heterotrimeric NF-Y complexes that may be combined in specific tissues. For example, CsNF-YA3/5, CsNF-YB6/7/9 and CsNF-YC5 could form potential NF-Y complexes in tissue of callus, while CsNF-YA2, CsNF-YB5/11 and CsNF-YC2/3 could do it in fruit tissue. Functional analysis using yeast two- or three-hybrid assays will help to clarify whether such interactions might be detected *in vitro*. Most *CsNF-Y* genes were also expressed in response to one or more stress treatments (Fig 3A), suggesting their involvement in the regulation of biotic and/or abiotic stress response. Those subunit members that were co-expressed in the same stress treatments may likewise represent potential NF-Y complexes regulating specific stress responses, such as *CsNF-YA5* and *CsNF-YB1/2/4/5/11* in drought and *CsNF-YA3* and *CsNF-YB3/6/9* in *X*. *fastidiosa* treatment. Interestingly, *CsNF-YA3* is an ortholog of the rice *OsHAP2E* gene, whose expression was shown to be induced after inoculation with *Magnaporthe oryzae* or *Xanthomonas oryzae* pv. *oryzae* and whose overexpression in transgenic rice resulted in enhanced resistance against these fungal and bacterial pathogens, respectively [30]. The similar expression profiles exhibited by the paralogous *CsNF-YB* genes in the different tissues (*CsNF-YB3/9* and *CsNF-YB8/10*; Fig 2) or stress treatments (*CsNF-YB3/9*; Fig 3A) suggest functional redundancy among these genes.

At phenotypic level, it has been well documented that sweet orange scions grafted on Rangpur lime typically exhibit impaired photosynthesis and enhanced root growth under water deficit treatment [62,63]. When these previous findings are considered together with our expression data (Fig 3B), in which *CsNFY-A5* is preferentially expressed in callus, a tissue showing high cell division rates, and up-regulated in roots of Rangpur but down-regulated in leaves of sweet orange by drought stress, we can assume its potential role as a candidate gene in the drought-induced regulation of the processes of root growth and photosynthesis. Although a number of NF-YB and NF-YC proteins have been identified as regulators of root growth [21,25,32] and photosynthesis [19,26,31,32] in different plant species, emerging evidences also supporting the role of NF-YA members in these processes have been reported more recently [30,57].

The overexpression of *CsNF-YA5* exerted distinct effects on the dehydration tolerance in leaf discs of transgenic tobacco, depending on the transgenic line (Fig 4A). These variations may be presumptively associated with the varying *CsNF-YA5* expression levels among the different transgenic lines (S8C Fig). It is possible that CsNF-YA5 has both activating and inhibitory effects on dehydration tolerance, depending on the expression levels of the protein or interacting proteins, or on expression of other intracellular and extracellular factors that have not yet been identified in the present study. On the other hand, the most obvious effects of *CsNF-YA5* overexpression were the inhibition of H_2_O_2_ production in dehydrated leaf discs as revealed by DAB staining (Fig 4B), improved biomass and root length as revealed by the *in vitro* drought stress tolerance assay (Fig 5A–5D), and decreased lipid peroxidation under drought treatment as revealed by TBARS analysis (Fig 5E). Such observations suggest a role of *CsNF-YA5* in plant growth and activation of the antioxidant system. Connections between NF-YAs and antioxidant defenses have been previously shown. Overexpression of the *AtNF-YA5* improved drought resistance in part by activating target antioxidant gene products, such as glutathione S-transferase, peroxidases and an oxidoreductase [4]. Overexpression of the *Setaria italica SiNF-YA1* enhanced drought and salt tolerance in transgenic tobacco by increasing the activity of superoxide dismutase (SOD), peroxidase (POD) and catalase (CAT) [60]. In addition, transient luciferase (LUC) expression assays showed that *SiNF-YA1* is able to activate the *LUC* gene driven by the tobacco *CAT* promoter [60].

Overexpression of *CsNF-YA5* significantly increased *A*, *gs* and *E* rates (Fig 6), which are important physiological determinants in increasing yields. A significant increase in net photosynthetic rate was also reported in transgenic rice lines overexpressing the *OsHAP2E* gene, which was correlated with the higher number of tillers and grain yield exhibited by the transgenic plants [30]. Studies have shown that NF-Ys exert a positive regulation on photosynthesis by inducing the expression of several genes related to this process, such as those coding for the small subunit of ribulose-1,5-bisphosphate carboxylase (RBCS) and the light-harvesting chlorophyll *a/b*-binding protein of photosystem II (CAB) [19], chloroplast ATP synthase [22,64], glutamyl-tRNA reductase (GluTR) and light-harvesting chlorophyll *a/b*-binding proteins associated with photosystem I (LHCA4 and LHCB4) [22].

In conclusion, the present study demonstrates that citrus NF-Y comprises a relatively small but diverse family of proteins involved in developmental processes and stress response. Functional analysis of *CsNFYA5* in transgenic tobacco plants evidenced its involvement in biochemical and physiological mechanisms of adaptation to drought that can contribute to the maintenance of plant growth and productivity in environments with short, frequent and moderate soil water deficit periods alternating with wet periods. The information presented here offers a useful foundation for functional studies of CsNF-Ys and supports the potential application of *CsNFYA5* in the improvement of drought tolerance in citrus via biotechnological strategies.

## Supporting information

S1 Fig Phylogenetic relationships of CsNF-YAs with members of the NF-YA family of *Arabidopsis thaliana*. The amino acid sequences were aligned using ClustalX and the Neighbor-Joining method. The tree was built with a bootstrap support of 1000 replications. Numbers at internal nodes denotes the results of bootstrapping analysis (n = 1000).
(TIF)

S2 Fig Phylogenetic relationships of CsNF-YBs with members of the NF-YB family of *Arabidopsis thaliana*. The amino acid sequences were aligned using ClustalX and the Neighbor-Joining method. The tree was built with a bootstrap of 1,000 replications. Numbers at internal nodes denotes the results of bootstrapping analysis (n = 1000).
(TIF)

S3 Fig Phylogenetic relationships of CsNF-YCs with members of the NF-YC family of *Arabidopsis thaliana*. The amino acid sequences were aligned using ClustalX and the Neighbor-Joining method. The tree was built with a bootstrap of 1,000 replications. Numbers at internal nodes denotes the results of bootstrapping analysis (n = 1000).
(TIF)

S4 Fig Chromosomal locations of *CsNF-Y* gene family. The chromosomal position of each *CsNF-Y* gene was mapped according to the *Citrus sinensis* Annotation Project (CAP). The *CsNF-YB8/10* pair of segmentally duplicated genes and *CsNF-YB3/9* pair of tandemly duplicated genes are indicated. The scale is in Mb.
(TIF)

S5 Fig Gene structure of *CsNF-Y* gene family members in *C*. *sinensis*. Open boxes correspond to 5’ and 3’ untranslated regions (UTR) and exons and introns are represented by filled boxes and black lines, respectively. The sizes of 5’ and 3’ UTRs, exons and introns can be estimated using the reference scale bar of 100 bp.
(TIF)

S6 Fig Amplification product by PCR using cDNA from Rangpur lime roots and specific primers for the amplification of *CsNF-YA5*. M: 1-kb molecular weight marker; C-: negative control (reaction without template cDNA); C+: positive control (reaction with plasmid DNA); N1 and N2: amplification product in Rangpur lime cDNA containing the expected size of approximately 846-bp.
(JPG)

S7 Fig *In vitro* regeneration of transgenic tobacco plants. Explants of tobacco in MS medium **(A)**. Initiation of the shoot formation in MS medium supplemented with BAP (5.0 mg L^-1^) and the antibiotics kanamycin (50 mg.L^-1^) and timentin (300 mg L^-1^) **(B, C)**. Plants developing in MS medium supplemented with kanamycin (50 mg L^-1^) and timentin (300 mg L^-1^) **(D)**. Individualized plants with the presence of roots **(E, F)**.
(JPG)

S8 Fig Molecular characterization of *CsNFYA5*-overexpressing transgenic lines. Amplification of the *nptII* gene fragment in transgenic tobacco plants by PCR **(A)**. M: 1-kb marker; C-: negative control (reaction without template DNA); C+: positive control (reaction containing plasmid DNA from pCAMBIA 2301); PC: transgenic line transformed with pCAMBIA 2301 empty vector; NT: non-transformed WT plants; N12-22: *CsNF-YA5*-overexpressing transgenic lines. PC DNA fragment shifted to an apparently higher molecular size due to the use of GelRed^™^ to stain the DNA. Histochemical assay for *uidA* gene expression in transgenic tobacco seedlings **(B)**. qRT-PCR expression analysis of *CsNF-YA5* in leaves of control (WT and PC) and *CsNFYA5*-overexpressing transgenic tobacco lines **(C)**. The data are means ± SE of three biological replicates in which *β-actin* (citrus) or *GAP2C* (tobacco) transcripts were used as internal controls. *, **Significantly different from WT at *P* ≤ 0.05 and *P* ≤ 0.01, respectively.
(TIF)
