# Supplementary figures and images for: Genome-wide characterization and expression analysis of citrus NUCLEAR FACTOR-Y (NF-Y) transcription factors identified a novel NF-YA gene involved in drought-stress response and tolerance
Source: PLoS One. 2018 Jun 15;13(6):e0199187. doi: 10.1371/journal.pone.0199187 (PMC6003680; doi:10.1371/journal.pone.0199187)

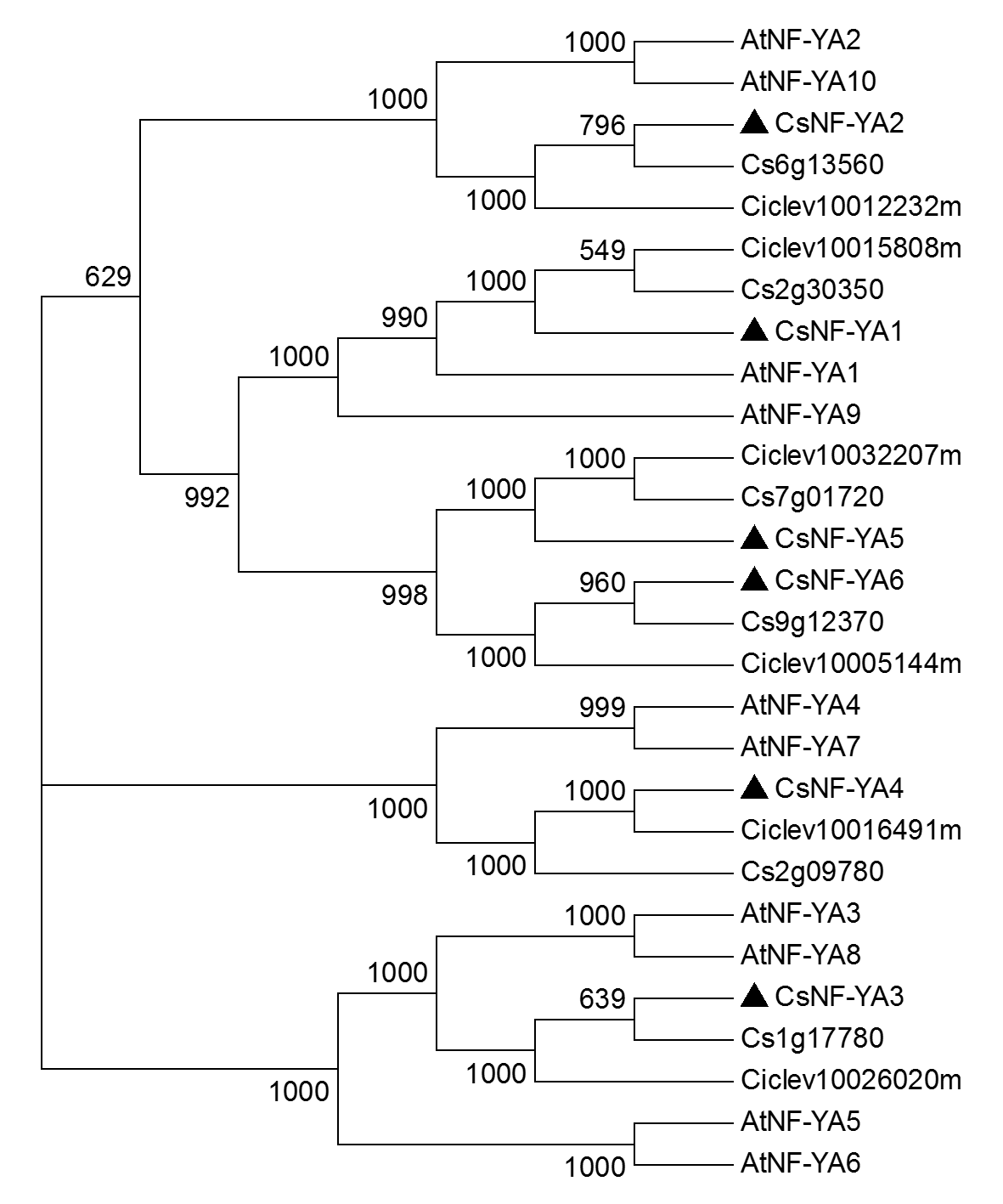

Supplement: S1 Fig — The amino acid sequences were aligned using ClustalX and the Neighbor-Joining method. The tree was built with a bootstrap support of 1000 replications. Numbers at internal nodes denotes the results of bootstrapping analysis (n = 1000). (TIF) [file pone.0199187.s001.tif]

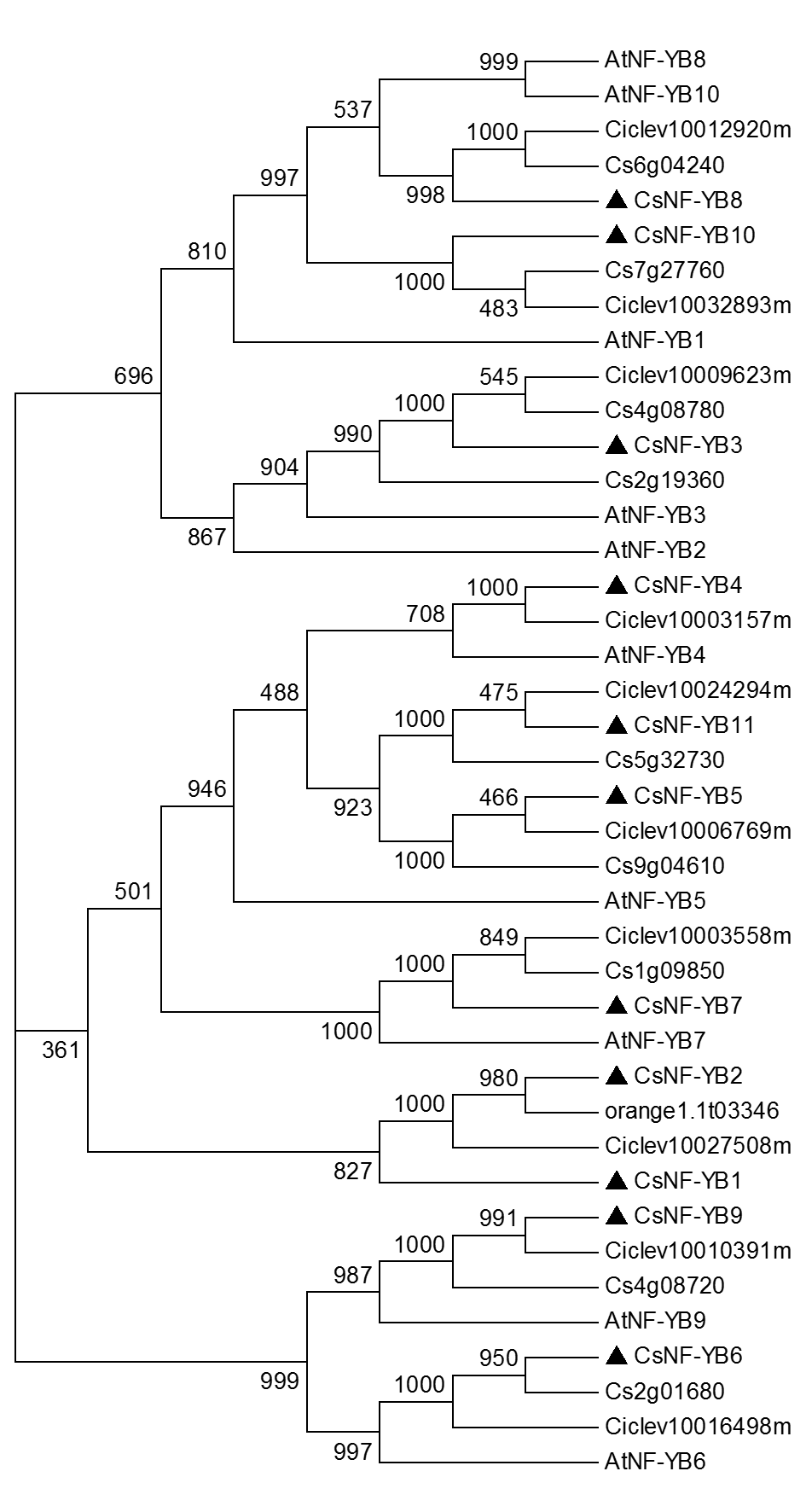

Supplement: S2 Fig — The amino acid sequences were aligned using ClustalX and the Neighbor-Joining method. The tree was built with a bootstrap of 1,000 replications. Numbers at internal nodes denotes the results of bootstrapping analysis (n = 1000). (TIF) [file pone.0199187.s002.tif]

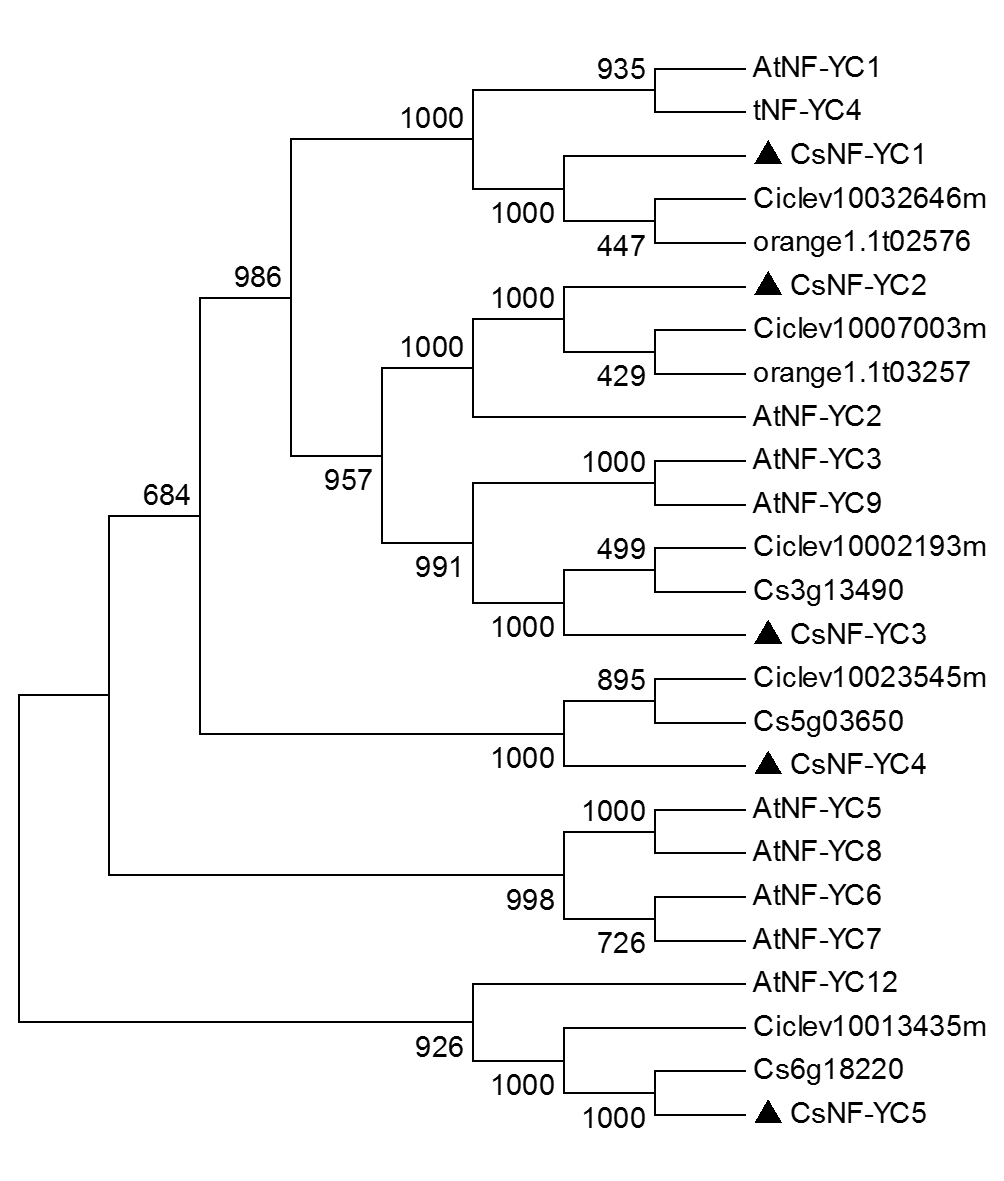

Supplement: S3 Fig — The amino acid sequences were aligned using ClustalX and the Neighbor-Joining method. The tree was built with a bootstrap of 1,000 replications. Numbers at internal nodes denotes the results of bootstrapping analysis (n = 1000). (TIF) [file pone.0199187.s003.tif]

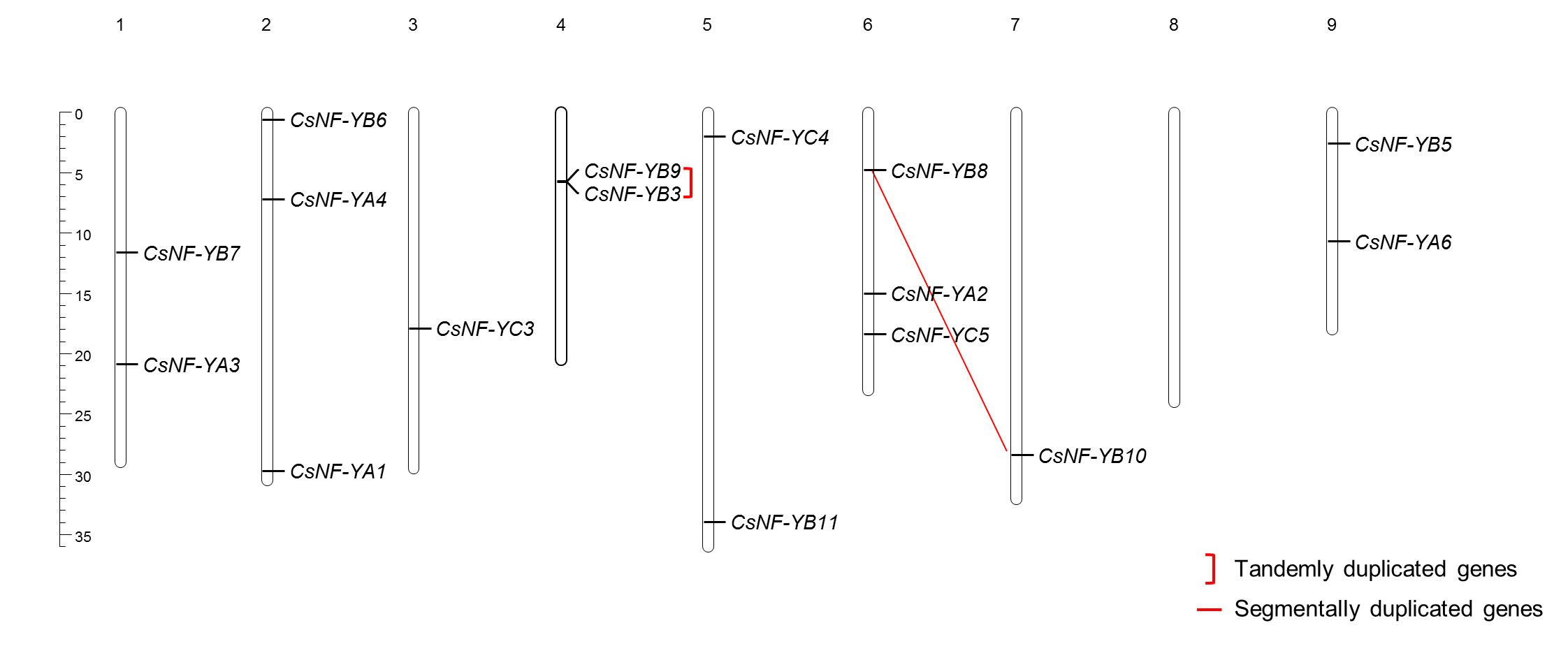

Supplement: S4 Fig — The chromosomal position of each CsNF-Y gene was mapped according to the Citrus sinensis Annotation Project (CAP). The CsNF-YB8/10 pair of segmentally duplicated genes and CsNF-YB3/9 pair of tandemly duplicated genes are indicated. The scale is in Mb. (TIF) [file pone.0199187.s004.tif]

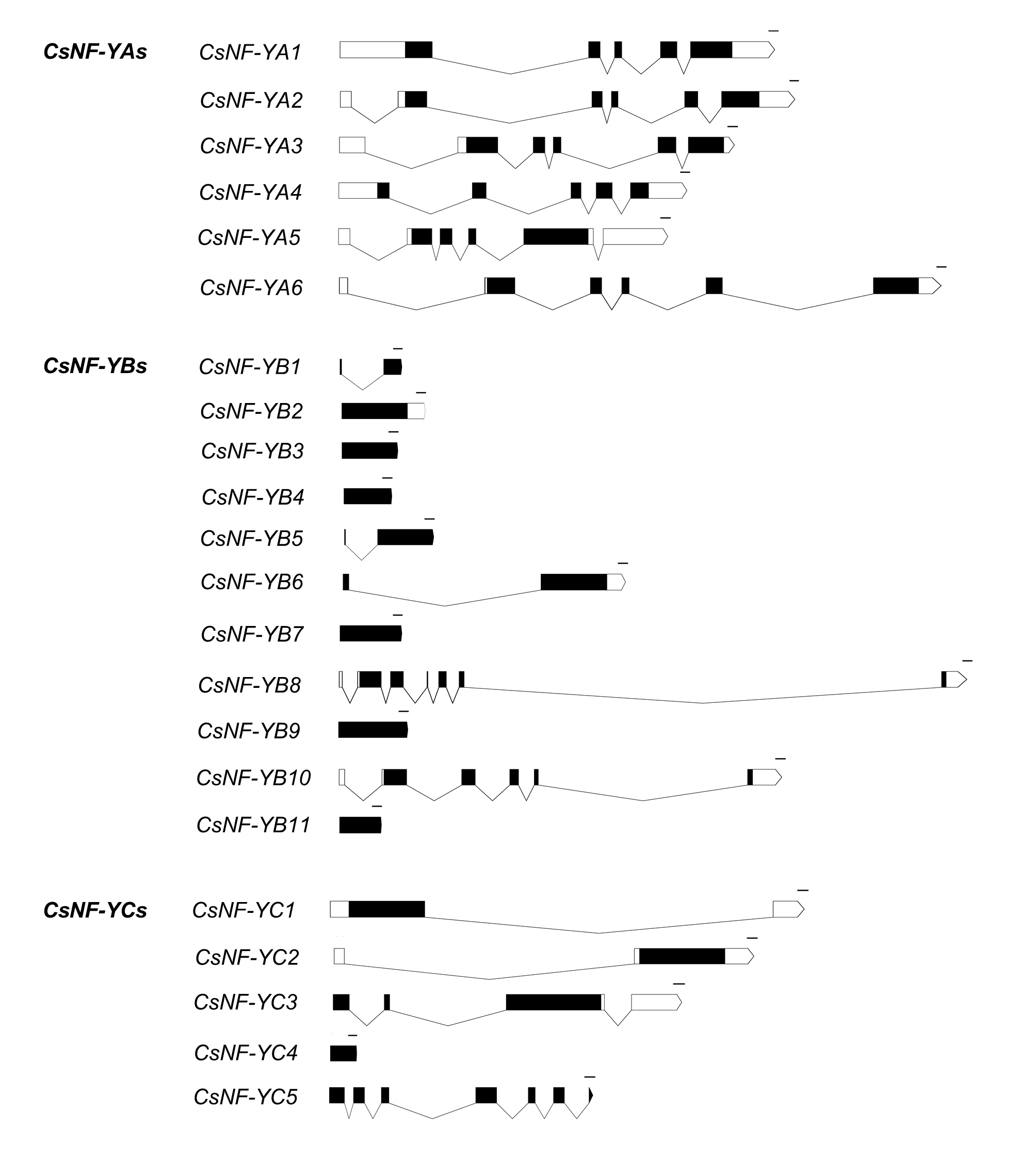

Supplement: S5 Fig — Open boxes correspond to 5’ and 3’ untranslated regions (UTR) and exons and introns are represented by filled boxes and black lines, respectively. The sizes of 5’ and 3’ UTRs, exons and introns can be estimated using the reference scale bar of 100 bp. (TIF) [file pone.0199187.s005.tif]

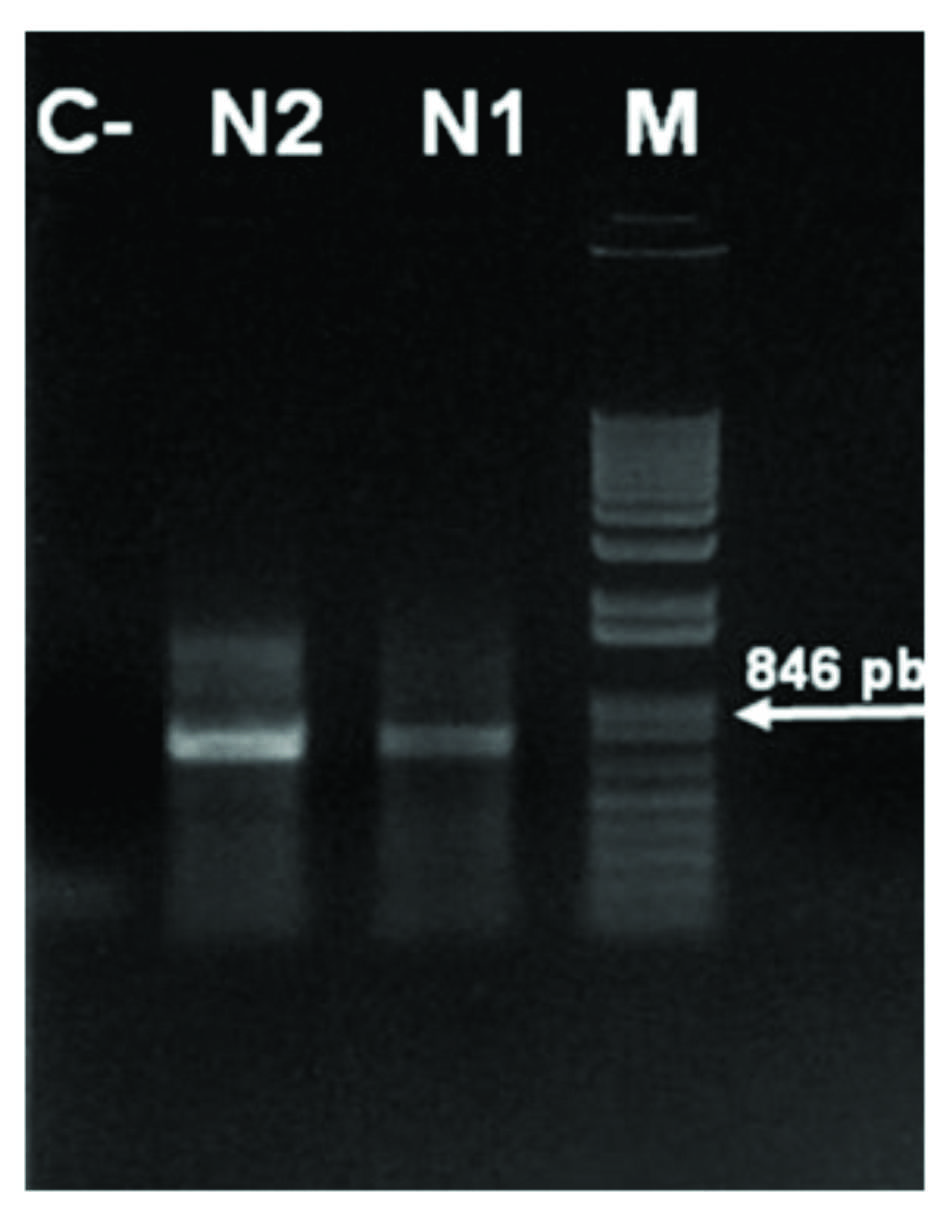

Supplement: S6 Fig — M: 1-kb molecular weight marker; C-: negative control (reaction without template cDNA); C+: positive control (reaction with plasmid DNA); N1 and N2: amplification product in Rangpur lime cDNA containing the expected size of approximately 846-bp. (JPG) [file pone.0199187.s006.jpg]

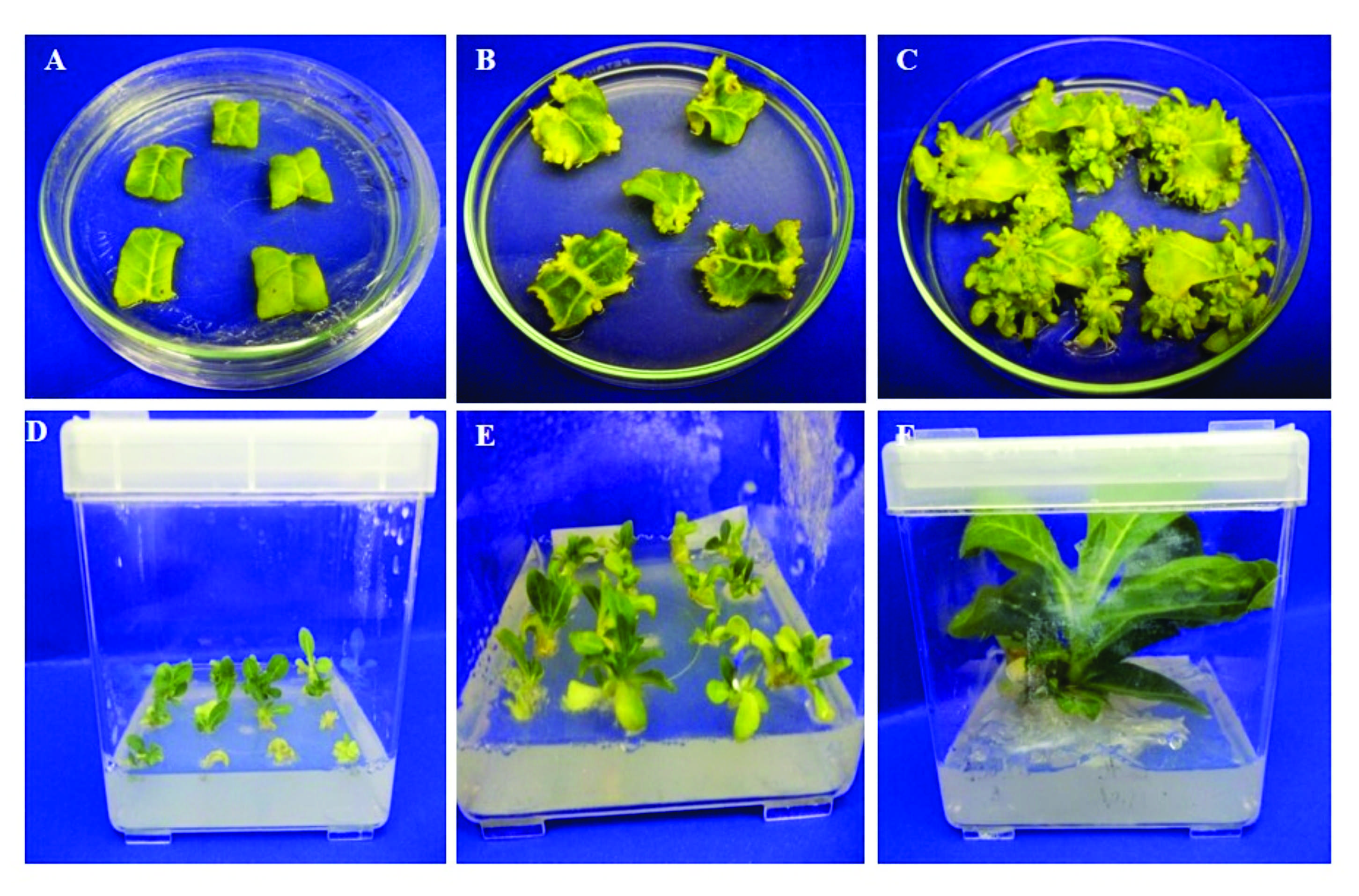

Supplement: S7 Fig — Explants of tobacco in MS medium (A). Initiation of the shoot formation in MS medium supplemented with BAP (5.0 mg L-1) and the antibiotics kanamycin (50 mg.L-1) and timentin (300 mg L-1) (B, C). Plants developing in MS medium supplemented with kanamycin (50 mg L-1) and timentin (300 mg L-1) (D). Individualized plants with the presence of roots (E, F). (JPG) [file pone.0199187.s007.jpg]

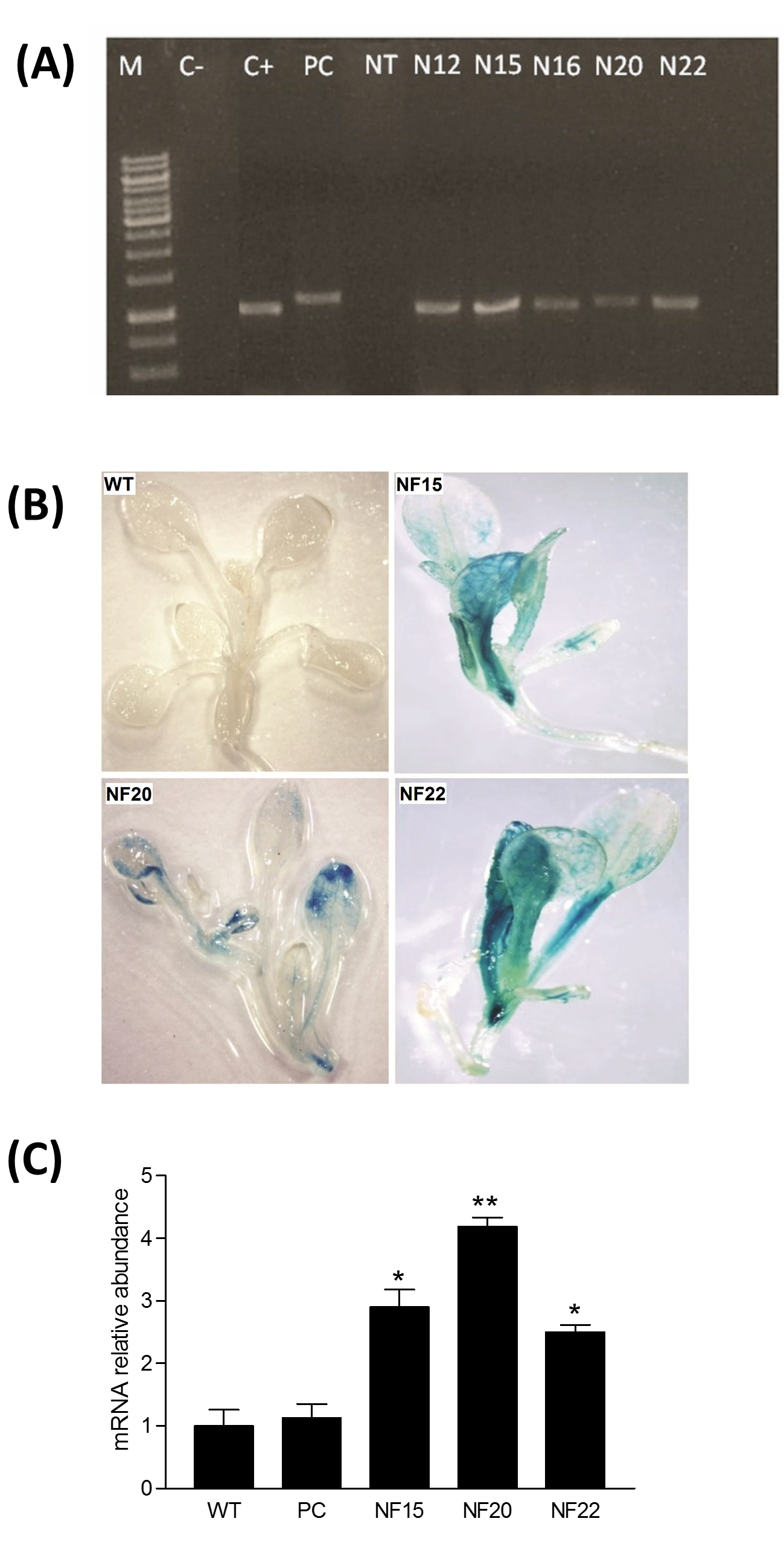

Supplement: S8 Fig — Amplification of the nptII gene fragment in transgenic tobacco plants by PCR (A). M: 1-kb marker; C-: negative control (reaction without template DNA); C+: positive control (reaction containing plasmid DNA from pCAMBIA 2301); PC: transgenic line transformed with pCAMBIA 2301 empty vector; NT: non-transformed WT plants; N12-22: CsNF-YA5-overexpressing transgenic lines. PC DNA fragment shifted to an apparently higher molecular size due to the use of GelRed™ to stain the DNA. Histochemical assay for uidA gene expression in transgenic tobacco seedlings (B). qRT-PCR expression analysis of CsNF-YA5 in leaves of control (WT and PC) and CsNFYA5-overexpressing transgenic tobacco lines (C). The data are means ± SE of three biological replicates in which β-actin (citrus) or GAP2C (tobacco) transcripts were used as internal controls. *, **Significantly different from WT at P ≤ 0.05 and P ≤ 0.01, respectively. (TIF) [file pone.0199187.s008.tif]
